# The metabolic genomic atlas reveals potential drivers and clinically relevant insights into the etiology of esophageal squamous cell carcinoma

**DOI:** 10.7150/thno.70814

**Published:** 2022-08-21

**Authors:** Xuesong Liu, Ruoxi Hong, Peina Du, Di Yang, Meibo He, Qingnan Wu, Lin Li, Yan Wang, Jie Chen, Qingjie Min, Jinting Li, Weimin Zhang, Qimin Zhan

**Affiliations:** 1 Department of Biochemistry and Biophysics, School of Basic Medical Sciences, Peking University Health Science Center, Beijing, China.; 2 Key Laboratory of Carcinogenesis and Translational Research (Ministry of Education/Beijing), Laboratory of Molecular Oncology, Peking University Cancer Hospital & Institute, Beijing 100142, China.; 3 Institute of Cancer Research, Shenzhen Bay Laboratory, Shenzhen 518107, China.; 4 Research Unit of Molecular Cancer Research, Chinese Academy of Medical Sciences, Beijing, China.; 5 Department of Oncology, Cancer Institute, Peking University Shenzhen Hospital, Shenzhen Peking University-Hong Kong University of Science and Technology (PKU-HKUST) Medical Center, Shenzhen 518035, China.; 6 Department of Medical Oncology, Sun Yat-Sen University Cancer Center, State Key Laboratory of Oncology in South China, Collaborative Innovation Center for Cancer Medicine, Guangzhou, Guangdong, 510060, P. R. China.; 7 BGI-Qingdao, BGI-Shenzhen, Qingdao, 266555, China.; 8 Institute of Systems Biomedicine, School of Basic Medical Sciences, Peking University Health Science Center, Beijing, 100191, China.; 9 SJTU-BGI Innovation Research Center, Shanghai, China. National Research Center for Translational Medicine, National Key Scientific Infrastructure for Translational Medicine (Shanghai), Shanghai Jiao Tong University, Shanghai, China.

**Keywords:** ESCC, metabolic genomic atlas, somatic mutations, copy number alterations (CNAs), clinical implications

## Abstract

**Background:** Esophageal squamous cell carcinoma (ESCC) is one of the most common cancers globally, with a poor prognosis and ambiguous therapy target. As a hallmark of cancer, metabolism reprogramming plays a critical role in the development of ESCC; however, the genomic alterations underlying this reconfiguration are still largely unknown.

**Methods:** We have comprehensively studied the metabolic genomic variations in an integrated ESCC cohort of 490 patients and characterized the somatic alterations associated with various metabolic pathways.

**Results:** The somatic mutations and copy number alterations (CNAs) occurred heterogeneously in all patients. Using CNA-based clustering, we stratified patients into three clusters and Cluster3 with more deletions marked for worse prognosis. Our findings revealed detailed genetic alterations in components of metabolic pathways and highlighted the role of metal ion channel transporters and non-neuronal/neuronal synapse systems in the development of ESCC. We found a subset of potential metabolic drivers and functionally validated RYR2, MGST3, and CYP8B1 involved in the ESCC-associated malignancy. Another key finding was that we identified 27 metabolic genes with genomic alterations that could serve as independent prognostic factors and figured out two genetic panels that could stratify patients into distinct prognostic groups.

**Conclusion:** Collectively, our study provided a deep insight into the metabolic landscape in ESCC, extending our understanding of the metabolic reconfiguration underlying the genomic basis of ESCC. Furthermore, our findings revealed potential prognostic factors of ESCC, which are expected to contribute to the accurate determination of the prognosis in the clinic.

## Introduction

Metabolic reprogramming is a hallmark of cancer 1. Almost a century ago, Otto Warburg demonstrated for the first time that tumor tissues consume more glucose than normal tissues and tend to depend on aerobic glycolysis 2. In the past decade, researchers have renewed their interest in cancer metabolism. Compelling pieces of evidence have supported the notion that altered metabolism contributes to tumorigenesis, not the other way around 3. Activated oncogenes (*e.g.*, RAS, MYC) and mutant tumor suppressors (*e.g.*, TP53, PTEN) drive the metabolic rewiring to support the rapid proliferation and survival of tumor cells 1. Although the energy generation and biomass production pathways - glucose, glutamine, and fatty acid metabolism - have been extensively studied 4, the complex landscape of oncogenic activity of metabolic pathways is far from understood. Therefore, investigating the roles played by various metabolic pathways in tumor development is urgently required.

Recent studies have identified candidate drivers of cancer metabolic adaptation by investigating the transcriptional deregulation in metabolic pathways across various cancers 5-7. Besides, the genomic alterations in metabolic genes responsible for tumorigenesis have been increasingly identified. The mutations in the genes of isocitrate dehydrogenase (IDH1/IDH2) 8-13 and succinate dehydrogenase (SDHA/SDHB/SDHC/SDHD) 14-21 families, and fumarate hydratase (FH) 22 were found in many types of human cancers. These alterations contributed to the rewiring of the catalytic pathways of these genes 3. These studies provided direct evidence that metabolic derangements could promote tumor progression; however, it is the tip of the iceberg to understanding the mechanisms of metabolic reprogramming in tumors. Recently, there have been several pan-cancer analyses of metabolic genomic and transcriptomic aberrances in different types of cancers 23-26. These studies extensively evaluated mutations, copy number alterations (CNAs), and transcriptional features of all metabolic genes and provided insights into the common core metabolic signatures across multiple tumor types. Although the common features of metabolism were determined using pan-cancer analysis, the study failed to elucidate the distinct metabolic characteristics of each tumor. Additionally, this analysis did not include esophageal squamous cell carcinoma (ESCC), the fourth leading cause of cancer-related mortality 27. The metabolic genetic landscapes of ESCC have not yet been investigated. Thus, a compelling need exists to generate higher resolution data and extensively identify precise somatic mutations and CNAs in ESCC. These data will increase our understanding of the genetic basis of the metabolic regulation networks and guide the development of therapeutic strategies for targeting cancer metabolism.

In this study, we conducted a joint analysis of our sequencing data and other ESCC sequencing cohorts and the genomic and transcriptomic data from The Cancer Genome Atlas (TCGA) to study the metabolic and genetic landscapes systematically. To our knowledge, this is the first study involving the largest cohort that characterizes the metabolic genomic landscapes of ESCC. We aimed to (i) determine whether somatic genetic alterations of metabolic genes prevalently occurred across different patients with ESCC and the extent to which such alterations were shared among multiple samples; (ii) determine whether distinct molecular subtypes exist in ESCC patients on a metabolic genetic basis; (iii) determine core metabolic drivers and the most affected metabolic pathways in ESCC; (iv) elucidate the clinical implications of such genetic variations. We also verified the expression levels and clinical association of the top-ranked altered gene (*RYR2*) in an independent ESCC cohort. Furthermore, we proved that potential driver metabolic genes (*RYR2, MGST3,* and* CYP8B1*) contributed to the malignant phenotypes of ESCC cells.

## Results

### Mutations in metabolic genes associated with ESCC

To understand the metabolic adaptation of ESCC on the genetic level, we integrated the data from the ESCC genomic cohort (490) obtained from seven previously published studies of ESCC 28. To specifically focus on metabolism, we used a previously curated list of 2752 metabolic genes encoding all known human metabolic enzymes and transporters from the Kyoto Encyclopedia of Genes and Genomes (KEGG) database (Table S1) 7.

We identified 9560 metabolic mutations with WES or WGS data from 490 ESCC patients in the present study, including 7235 non-silent and 2325 silent mutations (Table S2). The mutational types of metabolic genes were similar to the patterns of all mutations (Figure S1A). Different mutational processes often generate different combinations of mutational types, termed “signatures”. Our analysis revealed that C>T or G>A transition was the predominant type, which was present in the whole mutational spectrum (Figure S1B) 28. Next, a non-negative matrix-factorization (NMF) method was applied to explore the etiology of these mutational processes. As expected, this analysis revealed three mutational signatures that were consistent with the previous study (Figure S1C) 28. Signature1 characterized by C>T and C>G mutations in TpCpN trinucleotides was related to the over-activity of members of the APOBEC family of cytidine deaminases. Signature2 characterized by C>T mutations in NpCpG trinucleotides was associated with the spontaneous deamination of 5-methyl-cytosine. Finally, Signature3 characterized by the C > A mutations was hypothesized to be caused by tobacco mutagens. Using unsupervised hierarchical clustering of these three mutational signatures, we stratified the patients into three clusters (Figure S1C, Table S3); patients in Cluster 2 showed a worse prognosis than patients in Cluster 1/3 (Figure S1D). Taken together, these findings suggested that mutations of metabolic genes had similar etiology as overall mutations.

**Figure 1:**
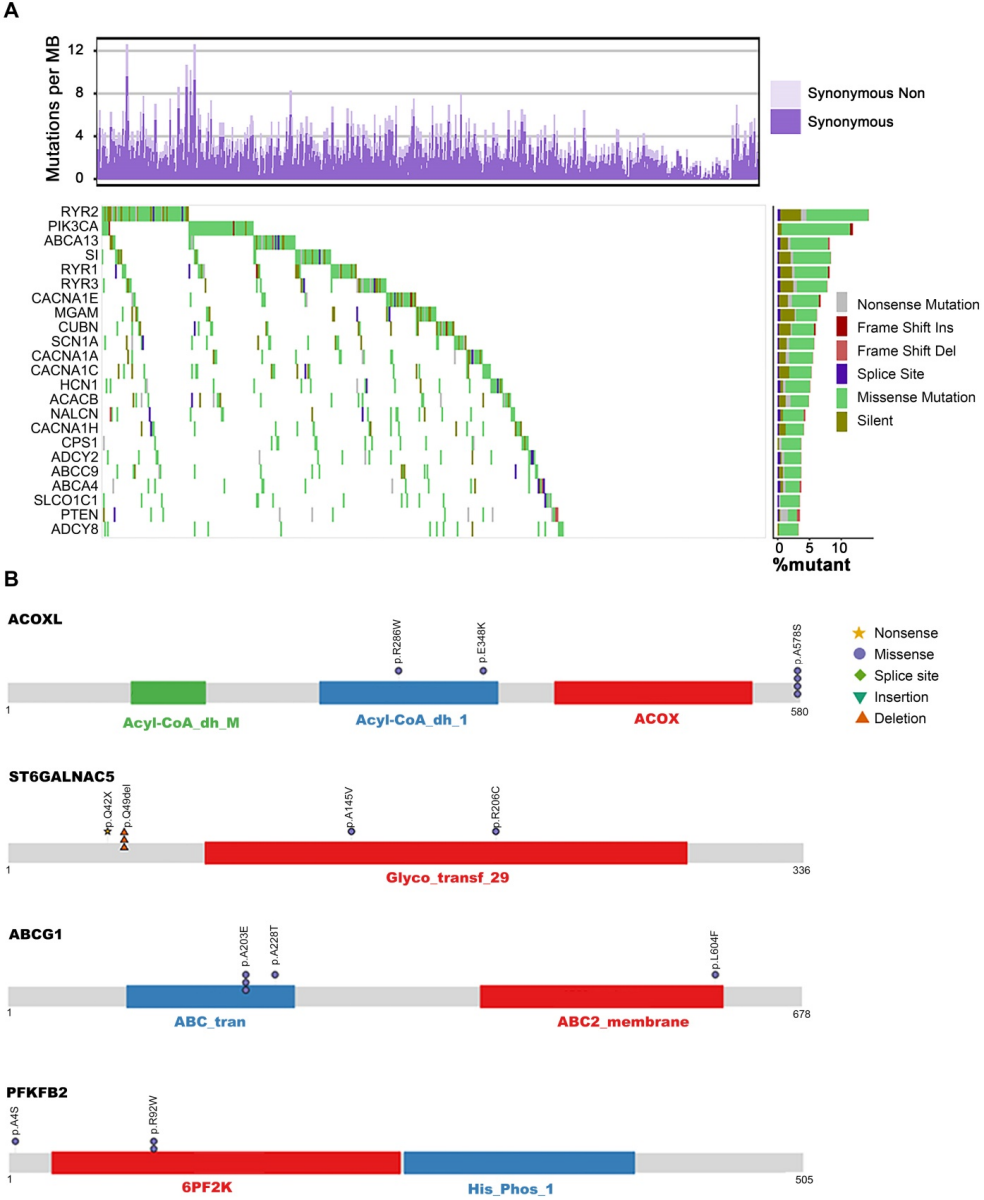
** The metabolic mutational landscape across 490 ESCC genomes. (A)** Top: number of synonymous and nonsynonymous mutations. Middle: mutated genes that were detected in more than 10 ESCC samples were colored by mutation types. Left: mutated genes ranked by mutation frequency. Right: Mutation frequency of each gene. **(B)** A schematic representation of the domain structure of proteins (ACOXL, ST6GALNAC5, ABCG1, and PFKFB2) encoded by recurrently mutated genes shows the location of recurrent residues identified in ESCC tumors.

Patients with ESCC harbored heterogeneous mutations in metabolic genes with counts ranging from 0 to 76 genes per patient and a median of 18 mutations. The counts of non-silent mutations ranged from 0 to 49 genes per patient with a median of 14 (Figure S1E, Table S4). From the viewpoint of recurrently mutated genes, we identified 23 metabolic genes with non-silent mutation frequency >3% (a gene at least mutated in 15 out of 490 patients) in our integrated ESCC cohort (Figure 1A). The mutated genes and the mutation frequencies are as follows: calcium signaling pathway (*RYR2*, 10.41%; *RYR1*, 5.71%; *RYR3*, 5.31%; *CACNA1E*, 5.31%; *CACNA1A*, 4.29%; *CANCNA1C*, 3.67%; *CACNA1H*, 3.06%; *ADCY2*, 3.47%; *ADCY8*, 3.06%), ATP binding cassette (ABC) transporters (*ABCA13,* 6.73%*; ACACB,* 3.88%*; ABCA4,* 3.27%*; SLCO1C1,* 3.27%; *ABCC9,* 3.06%), carbohydrate digestion and absorption pathways (*PIK3CA,* 10.82%*; SI,* 6.33%*; ACACB,* 3.88%*; NGAM,* 3.67%*; PTEN*, 3.06%) and other pathways (*HCN1,* 4.49%*; SCN1A,* 4.08%*; CUBN,* 3.88%*; NALCN,* 3.67%*; CPS1,* 3.47%). Except for the well-studied genes *PIK3CA* and *PTEN*, most of which have not yet been associated with ESCC, several genes are known to play critical roles in the development of tumors other than ESCC. The major members of the voltage-gated calcium channel (VGCC) family - *CACNA1A, CACNA1C, CACNA1E,* and *CACNA1H* - had highly frequent sporadic mutations in ESCC. These genes are also downregulated in cancers of the breast, kidney, lung, brain, and esophagus. Further, loss of expression of the *CACNA1C* gene results in rituximab resistance in diffuse large B-cell lymphoma 29-31*.*

It is well accepted that proteins encoded by genes with specific recurrent mutations are more likely to have biological functions in tumorigenesis and development 32. To this end, we sought to uncover the metabolic genes with recurrently mutated residues. Interestingly, we identified 72 genes with at least one specific mutated residue in more than two patients (Table S5). Using GO biological processes enrichment analysis, we found that most of these genes were enriched in potassium ion transport (*ATP1A4, KCND2, KCNMA1, NOS1, PTEN, KCNQ4, KCNH4, KCNK13, ALG10B, KCNT2, HCN1*), calcium ion transmembrane transport (*ATP2A1, GRIN1, NOS1, P2RX4, RYR2, RYR3, CACNA1H, TRPM7*), and sodium ion transport (*ATP1A4, NOS1, P2RX4, SCN5A, SLC6A3, CACNA1H, SLC23A2, SLC6A20, SCN3B, HCN1*). These findings suggest the significant involvement of metal ion channels in the progression of ESCC. Additionally, the genes of purine nucleotide biosynthesis (*ADCY8, FBP1, COX2, NOS1, PDE2A, PFKFB2, AK5, ADSS1, ADCY4*), amino acid metabolism (*GAD2, NOS1, PRODH, LGSN, ADSS1, SLC6A3, MTHFD1L, PFKFB2, PTEN, ENPP4, ACOXL, FBP1*), lipid biosynthesis (*PIK3CA, PTEN, RPE65, CACNA1H, ABCG1, SLC27A5, ETNPPL, ST6GALNAC5, ST6GALNAC3*), vitamin transport (*CUBN, SLC23A2, SLC2A10, MTHFD1L*), alcohol metabolism (*PTEN, RPE65, CUBN, CACNA1H, ABCG1, DHRS4*), glycolysis (*FBP1, PFKFB2*), and oxidative phosphorylation (*COX1, COX2*) were also significantly mutated in our analysis. Out of these significantly mutated genes, twelve - *RYR2, PIK3CA, ABCA13, SI, RYR3, MGAM, CUBN, HCN1, CACNA1H, ADCY2, ABCA4, PTEN,* and *ADCY8* - had recurrent mutations, confirming their involvement in ESCC (Figure 1A, Table S5). Although several genes harbored only a few mutations, they represented recurrent residue enrichment phenomenon: *ACOXL* (p.A578S, 4/6)*, ST6GALNAC5* (p.Q49del, 3/6)*, ABCG1* (p.A203E, 3/5), *PFKFB2* (p.R92W, 2/4. Figure 1B)*, ABCE1* (p.Y350N, 2/4),* FBP1* (p.L257V, 2/4)*, SLC7A13* (p.L388V, 2/5)*, SCN3B* (p.S86R, 2/2)*, MT-CO2* (*COX2*, p.V142I, 2/5)*, LIPN* (p.G210D, 2/3)*, GRIN1* (p.E619del, 2/3)*, ADSSL1* (p.D76N/V, 2/3), *ATP2A1* (p.S383T/F, 2/3), *B4GALT2* (p.S65del, 2/2), *GCNT4* (p.R166S/H, 2/4), *UGT1A5* (p.A81V, 2/3) and *ADCY4* (p.L34del, 2/5. Table S5, Figure S2). These findings suggested the importance of these recurrently mutated residues in ESCC development; however, most of these residues are not linked to ESCC yet. Therefore, further studies are required to elucidate the biological functions of these recurrent mutations, which may uncover novel ESCC-associated metabolic drivers and potential therapeutic targets.

### *RYR2* is associated with malignancy in ESCC

**Figure 2:**
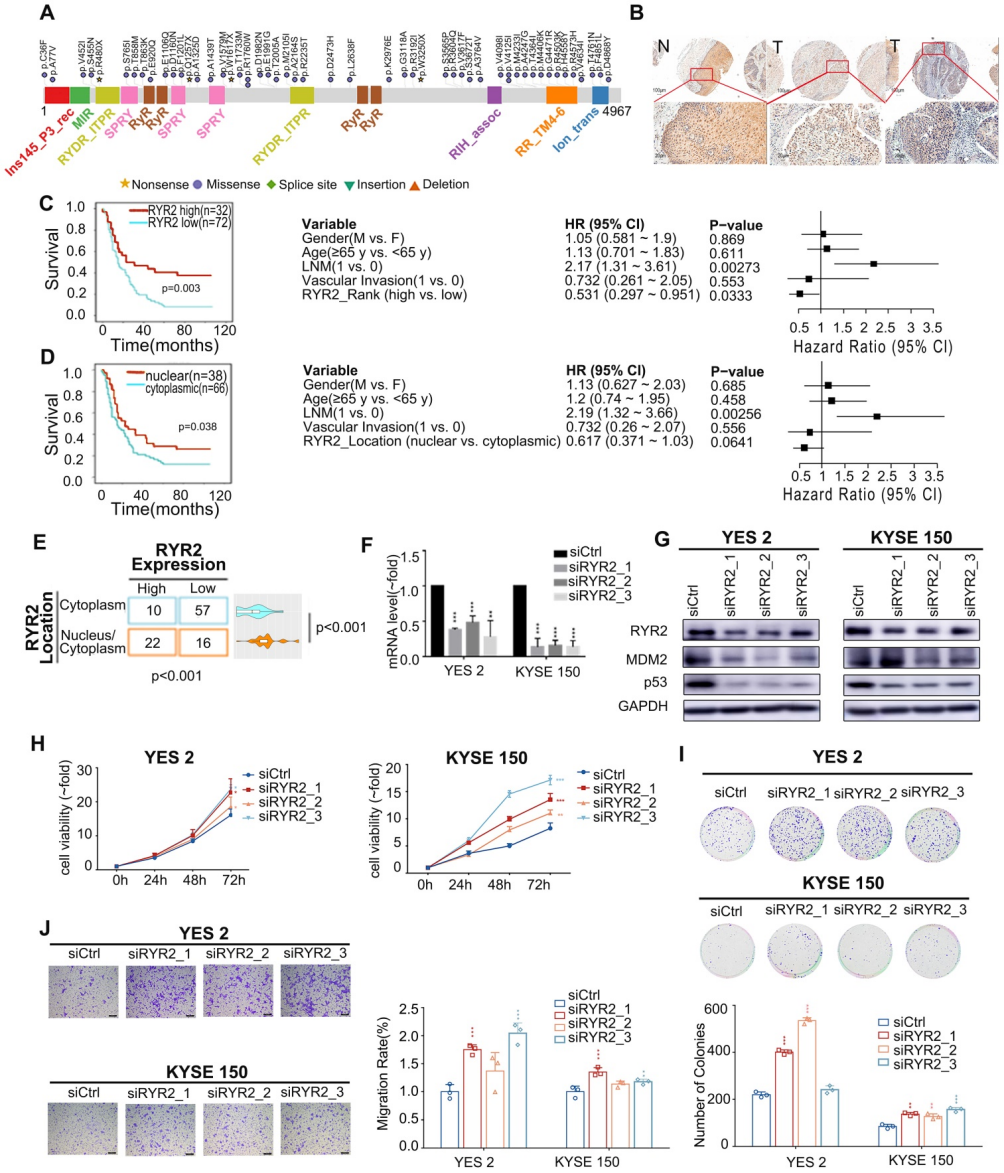
** Deficient RYR2 contributes to malignant phenotypes of ESCC. (A)** A schematic representation of the domain structure of RYR2 shows the location of somatic variants identified in ESCC tumors. **(B)** Representative images of IHC for RYR2 in ESCC tissues and matched adjacent normal tissues. Scale bars, 200 µm. **(C)** Left: Kaplan-Meier survival analysis of patients with ESCC stratified by RYR2 expression (n = 104; p = 0.003, log-rank test). Right: Multivariate analysis of the hazard ratios (HR) showed that the downregulation of RYR2 was an independent prognostic factor for the overall survival (by the multivariate Cox proportional hazard regression model). The HR is presented as the means (95% confidence interval, 95% CI). **(D)** Left: Kaplan-Meier survival analysis of patients with ESCC stratified by RYR2 localization (n = 104; p = 0.038, log-rank test.) Right: Multivariate analysis of the hazard ratios (HR) showed that the translocation of RYR2 may be an independent prognostic factor for the overall survival rate (by the multivariate Cox proportional hazard regression model). The HR is presented as the means (95% confidence interval, 95% CI). **(E)** RYR2 expression and localization in normal and tumor tissue. **(F)** The mRNA levels of RYR2 in YES2 and KYSE150 cells after a knockdown by siRNAs. **(G)** The protein levels of RYR2, MDM2, and p53 in YES2 (left) and KYSE150 (right) cells after a knockdown by siRNAs. **(H)** MTS assay on YES2 and KYSE150 cells transfected with siRYR2 or siCtrl. **(I)** Colony formation assay on YES2and KYSE150 cells transfected with siRYR2 or siCtrl. Up: representative images. Down: statistical analysis. **(J)** Transwell assays on YES2 and KYSE150 cells transfected with siRYR2 or siCtrl. Left: representative images. Right: statistical analysis.

Our data revealed highly frequent mutations in ryanodine receptors (RYRs) - *RYR1, RYR2,* and *RYR3* in ESCC patients, out of which several were truncating mutations (Figure 2A, S3A). Interestingly, *RYR2* mutation was the most prevalent alteration (64 cases, 13.1%). The most common type of *RYR2* mutation was a missense mutation (10.41%, Figure 1A, 2A). RYRs are significant Ca^2+^ mobilization channels responsible for the rapid release of Ca^2+^ from the sarcoplasmic/endoplasmic reticulum (SR/ER) into the cytoplasm; these functions are critical for cell proliferation, apoptosis, autophagy, and migration 1, 4, 33. However, the biological functions of RYRs in tumorigenesis are largely unknown.

To further explore the biological functions of RYR2 in ESCC, we first detected the expression of RYR2 in an independent ESCC cohort using the IHC assay (Table S6). The expression of RYR2 was remarkably decreased in tumor tissues than in the adjacent normal tissues (Figure 2B). We further explored the association between expression level/subcellular localization of RYR2 and clinicopathological features of patients with ESCC. We found a significant negative correlation between RYR2 expression and overall patient survival (0.005, Table S7). Importantly, Kaplan-Meier survival analysis also reported that RYR2 downregulation was strongly associated with poor survival of ESCC patients (p = 0.003, log-rank test, Figure 2C, left); the 5-year survival rate in the high RYR2 expression group (30.77%) was substantially lower than that in the low RYR2 expression group (69.23%). The median survival in the high RYR2 expression group was 23 months (0.000 to 57.648), while that in the low RYR2 expression group was 15 months (10.844 to 19.156). Multivariate Cox regression survival analysis adjusted for age, LNM, gender, and RYR2 level consistently highlighted a strong correlation between RYR2 expression and survival (p = 0.0333, HR = 0.531, 95% CI 0.297 to 0.951, Figure 2C, right). These observations indicated that RYR2 expression is an independent prognostic factor for the outcomes of patients with ESCC. Additionally, the localization of RYR2 also varied in tumor tissues. RYR2 tended to express more in the cytoplasm, while only a subset of tumor tissues had evident nuclear staining of RYR2 (Figure 2B, 2E). Interestingly, tumor tissues with cytoplasm and nuclear staining of RYR2 showed a higher expression level of RYR2 than the tissues with only cytoplasmic expression (0.001, Figure 2E). Patients with cytoplasmic and nuclear expression of RYR2 had a significantly better prognosis (Figure 2D, left). Using COX regression analysis adjusted for age, LNM, gender, and RYR2 localization status, we found a moderate prognostic value of RYR localization status (Figure 2D, right).

To have independent validation of the finding, we assessed the association of RYR2 expression levels with patient survival in an independent cohort. We obtained RYR2 protein expression levels and corresponding patient clinical information from a published article 34. In this independent cohort (hereafter termed “Proteomics Cohort”), the authors performed protein TMT mass spectrometry to quantify 124 pairs of ESCC tumor tissues and paired adjacent normal tissues. We analyzed the relationship between RYR2 expression and overall survival (OS). As expected and consistent with our results, patients with low RYR2 expression levels had a poorer survival prognosis (p = 0.065, Figure S4). Although the p-value is not significant, the trend is consistent. Collectively, these results suggest that the expression and localization of RYR2 might affect its biological functions and that RYR2 might be associated with tumor progression.

Since RYR2 is a huge gene with sporadic mutations and a lower expression in tumor tissues than adjacent tissues in ESCC, it is difficult to carry out ectopic overexpression. Thus, we explored the biological functions of RYR2 by interfering with its expression. We knocked down *RYR2* in ESCC cell lines - YES2 and KYSE150 - by RNAi technology. The mRNA and protein levels of *RYR2* demonstrated its decreased expression achieved using siRNAs (Figure 2F-G). MTS and colony formation assay findings suggested that the silencing of *RYR2* promoted cell growth (Figure 2H-I). Furthermore, the Migration assay demonstrated that the migration abilities also increased after* RYR2* down-regulation (Figure 2J). Together, the findings suggested that the loss of RYR2 function promotes the malignant phenotypes of ESCC.

**Figure 3:**
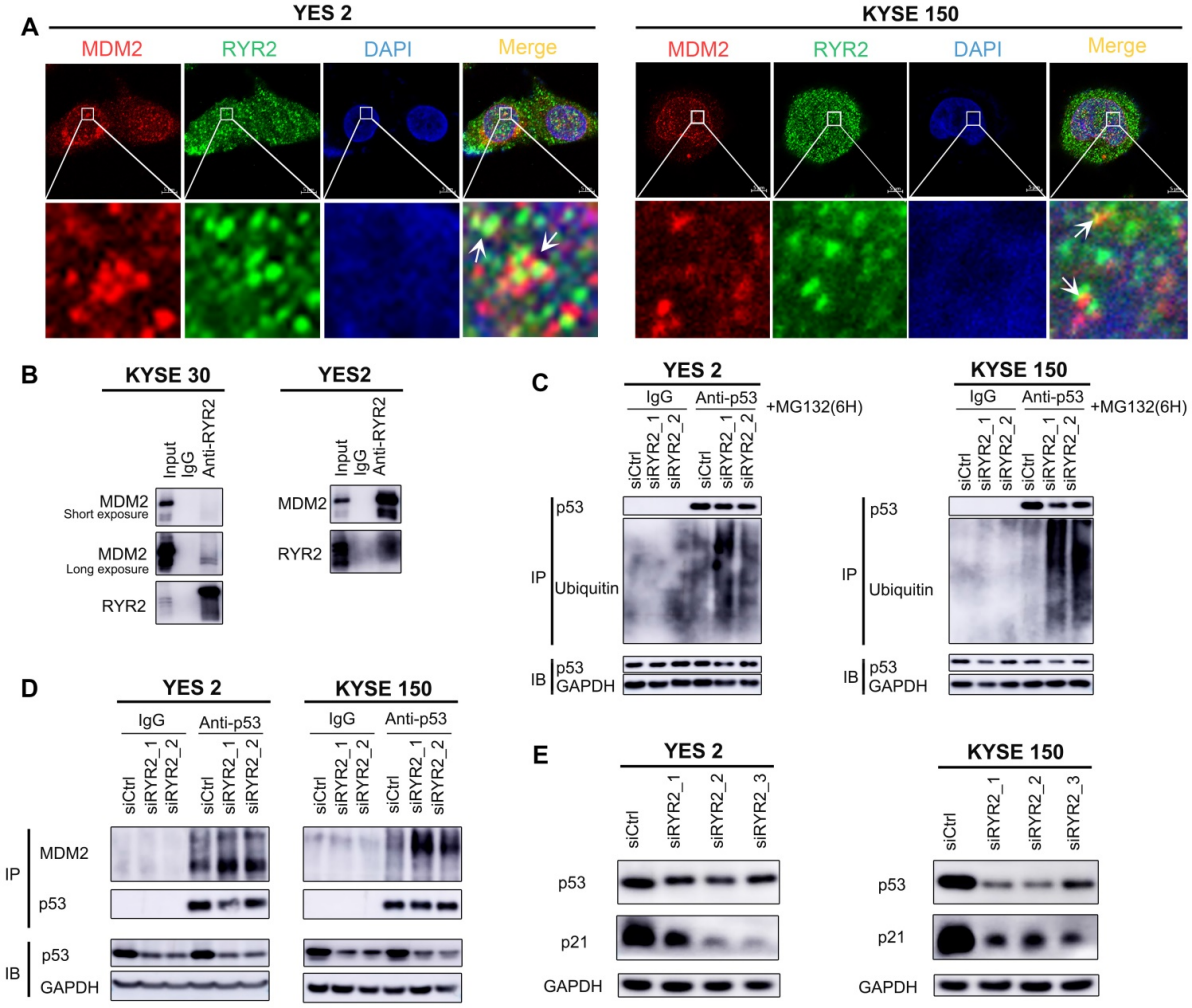
** RYR2 interacted with MDM2. (A)** Immunofluorescence analysis of the localization of RYR2/MDM2 in YES2 and KYSE150 cells. **(B)** Co-immunoprecipitation of RYR2 and MDM2 by RYR2 antibody. The cellular lysate was used to monitor the expression of MDM2 and RYR2 by western blotting (input). From the remaining cellular lysate, RYR2 was immunoprecipitated and associated MDM2 was detected by western blotting. Precipitation with IgG was performed as a negative control. **(C)** Ubiquitination of endogenous p53 was analyzed by immunoprecipitation with p53 antibody and followed by western blot analysis. YES2 and KYSE150 cells were knockdown of RYR2 and after 42h the cells were treated with MG132 (10 µM) for another 6h, then the cells were lysis and used for IP assay. **(D)** Co-immunoprecipitation of p53 and MDM2 by p53 antibody. YES2 (left) /KYSE150 (right) cells were lysed and used to monitor the expression of MDM2 and p53 by western blotting (input). From the remaining cellular lysate, p53 was immunoprecipitated and associated MDM2 was detected by western blotting. **(E)** Western blot analysis of the expression level of p53, p21 and GAPDH in YES2 and KYSE150 cells transfected with siRYR2 or siCtrl for 48h.

We then investigated the mechanisms underlying these malignant phenotypes. RYR2 was predicted to physically interact with MDM2 (Integrated Interactions Database, http://iid.ophid.utoronto.ca/). MDM2 binds to the transactivation domain of p53 and mediates its ubiquitination and degradation 35. Since nuclear RYR2 expression was associated with a better prognosis (Figure 2B), we were curious whether RYR2 could competitively interact with MDM2 and thus protect p53 from MDM2-mediated degradation. To this end, immunofluorescence assays were performed in YES2, KYSE150, and nine other cell lines to study the co-localization of RYR2 and MDM2. The findings revealed that RYR2 and MDM2 co-localize in the nucleus (Figure 3A, S3B). In agreement with these observations, RYR2 was co-precipitated with MDM2 (Figure 3B). As expected, the knockdown of RYR2 significantly increased the ubiquitination level of p53 (Figure 3C) and enhanced the interaction between p53 and MDM2, promoting the degradation of p53 (Figure 3D). Additionally, the knockdown of RYR2 also led to the decreased expression of p21, the classical downstream target of p53 (Figure 3D-E).

Collectively, our findings uncovered a novel mechanism in which RYR2 competitively interacts with the E3 ubiquitin ligase MDM2 and protects p53 from MDM2-mediated degradation. However, RYR2 was frequently downregulated in ESCC tumor tissues, leading to the decreased protein levels of p53 and p21 and promoting malignancy in ESCC.

### Copy number alterations of metabolic genes in ESCC

**Figure 4:**
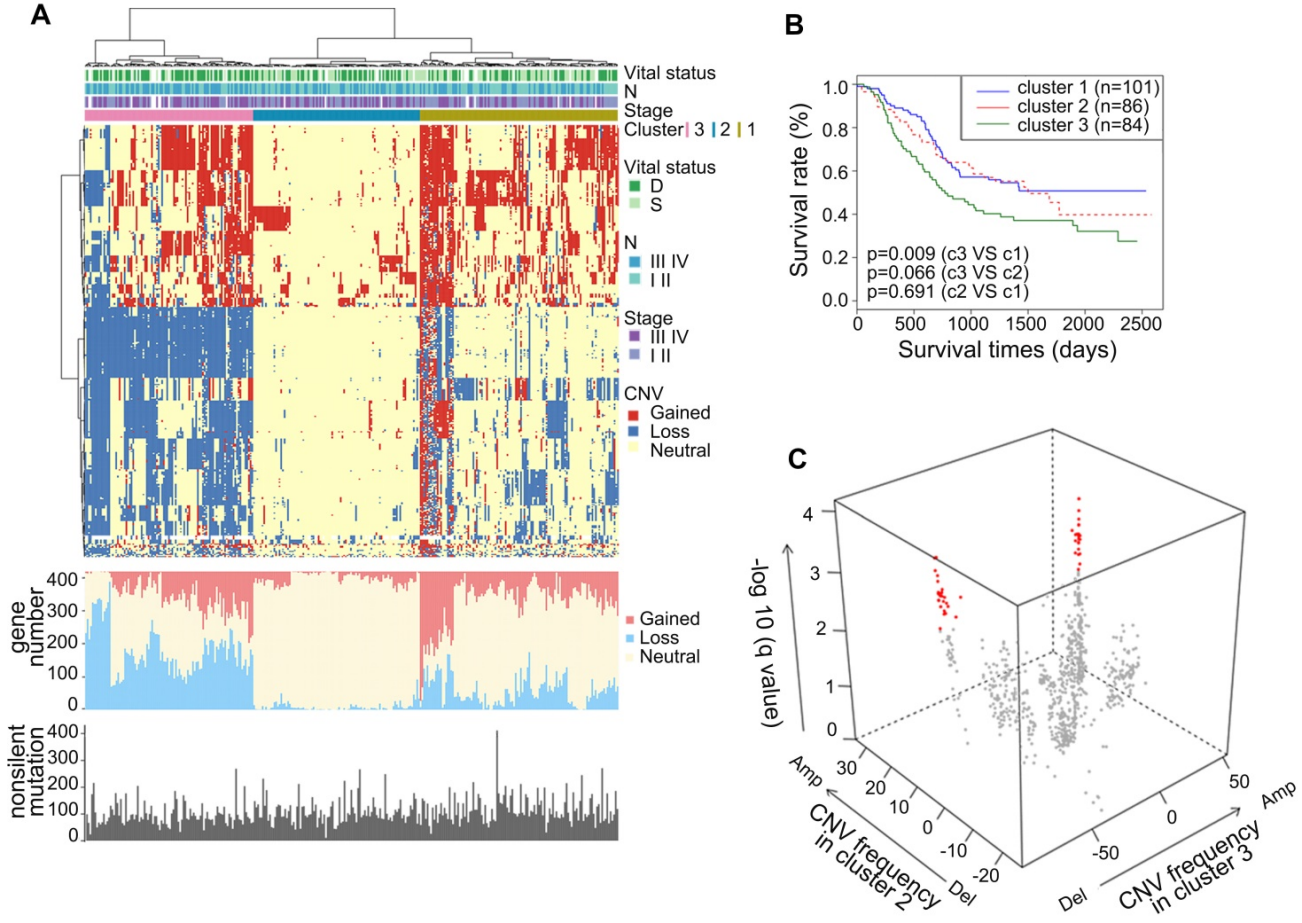
** Somatic copy number alterations analysis in integrated ESCC cohort and characterization of different subtypes. (A)** Hierarchical clustering analysis on the CNA of 420 top rank frequent alterations metabolic-related gene. Upper bars: stage, lymphatic metastasis, and vital status. Bottom bars: nonsilent mutations of each sample and number of CNAs. **(B)** Kaplan-Meier analysis comparing survival of patients stratified by different clusters. **(C)** Multidimensional scaling screen for metabolic-related genes by comparing subtypes 3 and 2. Genes that q < 0.001 and frequency of copy number gain or loss in subtypes 3 or 2 ≥ 20% were highlighted in red.

To gain comprehensive insights into the CNAs of metabolic genes in ESCC, we analyzed the CNAs in the metabolic genes from our integrated ESCC cohort (314). Genes harbored in focused regions with the frequency of copy number gain or loss ≥25% were selected for further analysis. We found 420 metabolic genes with such CNA frequency. Using hierarchical clustering analysis on the selected metabolic genes, we stratified the patients of the integrated ESCC cohort into three clusters (Figure 4A). Patients in Cluster1 exhibited more amplification while Cluster3 exhibited more CNA loss. The average amplification/gain frequency in the three clusters were 21.56%, 5.80%, and 16.88%, respectively, and the average deletion/loss frequencies in the three clusters were 13.86%, 2.04%, and 42.57%, respectively. There were no apparent differences in the non-silent mutations of these three clusters (Figure 4A). Kaplan-Meier analysis indicated that the patients of Cluster3 had a worse prognosis than patients of Cluster1 (p = 0.009, Figure 4B). To determine the discriminatory genes of these three clusters, we performed Student's t-test with FDR correction between each cluster pair. Only Cluster2 and Cluster3 had significant genes with different CNA statuses. These genes included 33 essential differential genes with loss frequency >80% in Cluster3 and a low frequency of loss in Cluster1 (q < 0.001, Figure 4C, Table S8). Interestingly, the most significant discriminatory gene, *FHIT,* was lost in 96.97% of Cluster3 (Table S8). This gene encodes a protein named Fhit, an enzyme of the histidine triad family that catalyzes the degradation of nucleoside 5',5'-triphosphates. This is regarded as the genome “caretaker”, and loss of Fhit causes nucleotide imbalance, spontaneous replication stress, and DNA breaks 36. The high frequency of *FHIT* loss in Cluster3 indicated its role in the ESCC development. Among the top 10 relevant genes, *ACOX2* and *ACAA1* participate in acyl-CoA metabolism; *PDHB* is a subunit of pyruvate dehydrogenase E1; *SCN5A* is a sodium voltage-gated channel subunit having recurrent mutation residue (Table S5, S8).

In terms of CNA frequency, the top 10 most frequent gain metabolic genes were *SQLE* (40.13%), *ADCY8* (39.81%), *TPCN2* (39.49%), *NDUFB9* (39.49%), *KCNQ3* (38.54%), *NCEH1 (*38.54%), *PIK3CA* (38.22%), *PLD1* (38.22%), *SLC7A14* (37.90%), and *SLC2A2* (37.90%), while the top 10 most frequent loss metabolic gens were *GXYLT2* (41.08%), P*DE12* (40.76%), *GPX1* (39.17%),* SLC25A20* (38.85%),* GBE1* (37.90%), *MTAP* (37.90%), *IP6K2* (37.26%), *ABHD6* (36.94%), *TKT* (36.62%), and *SLC30A5* (36.62%). Among these CNA-affected genes, *ADCY8, KCNQ3,* and *PIK3CA* harbored more than 2.00% non-silent mutations (Table S2). CNA is one of the mechanisms that affect mRNA expression. Thus, we investigated whether these CNAs were significantly associated with mRNA expression. Interestingly, we found that about half of the top 10 most frequent gain and loss genes significantly correlated with the mRNA level and CNA in the TCGA ESCC cohort (0.001, Pearson's Correlation Coefficient > 0.4, Table S9). These genes included CNA gain genes - *SQLE, ADCY8, TPCN2, NDUFB9, KCNQ3, NCEH1,* and *PIK3CA* and CNA loss genes - *PDE12, SLC25A20,* and *MTAP*. These findings highlighted the possible roles of these genes in the tumorigenesis of ESCC. The most amplified gene,* SQLE*, encoding for squalene epoxidase, is one of the key enzymes in the later stages of cholesterol synthesis. *SQLE* is also amplified in other solid tumors and contributes to malignancy. Targeting of SQLE by a small-molecule NB-598 raises the possibility that SQLE can be targeted in ESCC 25.

### Genetic heterogeneity of metabolic pathways in ESCC

**Figure 5:**
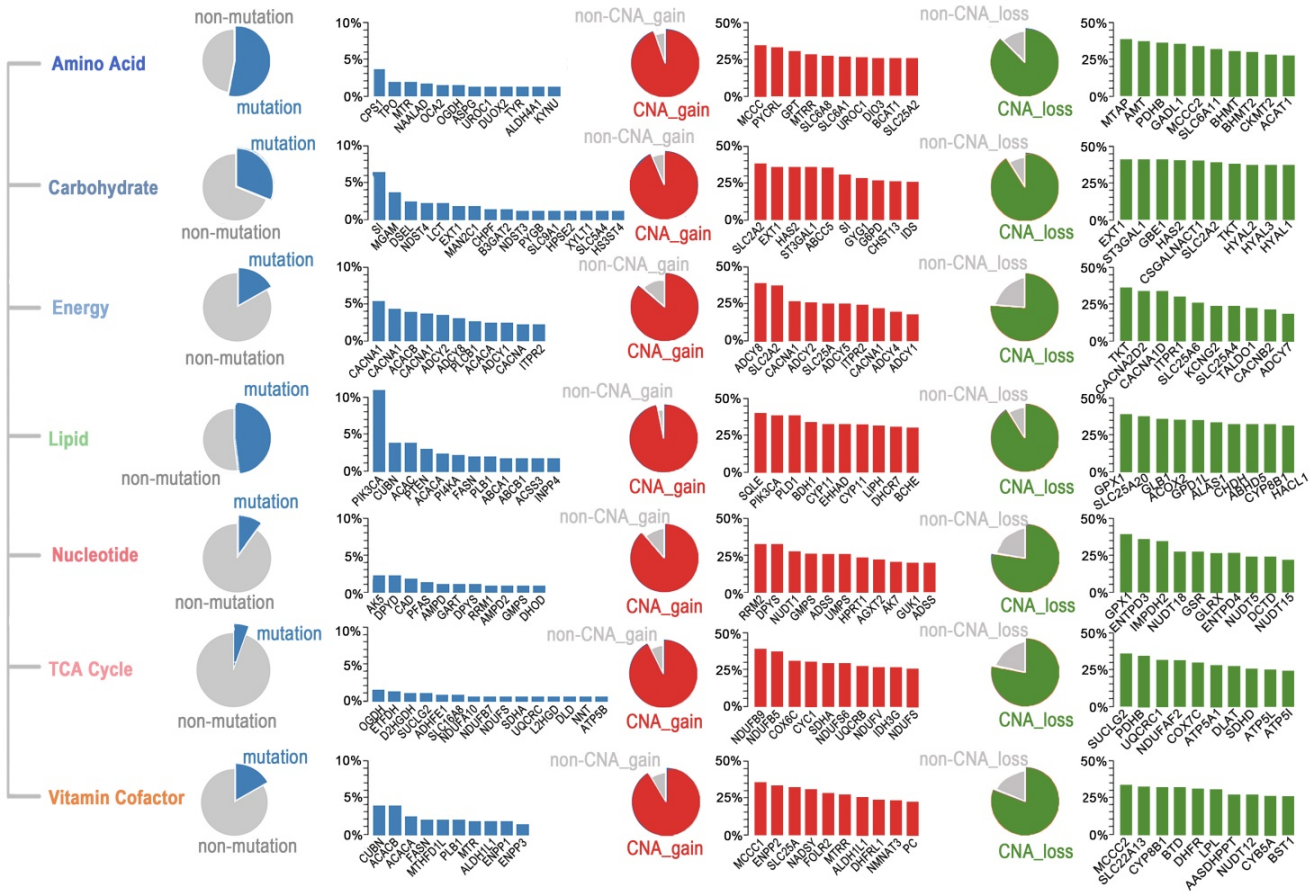
Somatic mutations and CNAs in seven major metabolic super-pathways reveal genetic heterogeneity of metabolic pathways in ESCC. The sector graph and histogram represented the mutation frequency of the pathway and the indicated genes, respectively.

To determine which metabolic pathways are significantly affected in ESCC, we first studied the metabolic genes of the seven major metabolic processes curated by Xinxin Peng, which included amino acid metabolism, carbohydrate metabolism, integration of energy, lipid metabolism, nucleotide metabolism, tricarboxylic acid cycle (TCA cycle), and vitamin and cofactor metabolism 26. The mutational rates of genes in these seven metabolic super-pathways were more heterogeneous than those affected by CNAs (Figure 5). Notably, the amino acid super-pathway harbored the highest number of mutations, followed by lipid, carbohydrate, and vitamin cofactor super-pathways. Mutations in the nucleotide and TCA cycle super-pathways were less prevalent (Figure 5). In the amino acid super-pathway, CPS1 (carbamoyl phosphate synthase 1), a urea cycle enzyme, had the highest mutation rate, a trend also observed in other tumors (Figure 5). CPS1 is reported to crosstalk with KRAS/LKB and EGFR pathways and maintain pyrimidine pools and DNA synthesis, promoting cell proliferation 37, 38. Several CPS1 inhibitors are developed, having potential in clinical practice 39, 40. As per a few studies, dysregulated lipid metabolism is an important characteristic of ESCC; however, detailed investigation in a larger cohort is still needed 41. In our integrated ESCC cohort, the top ten mutations in lipid super-pathway included mutations in kinase *PIK3CA* and* PI4KA*, intrinsic factor-vitamin B12 receptor *CUBN,* phosphatase *PTEN,* Phospholipase* PLB1*, acetyl-coenzyme A carboxylase* ACACA* (also known as *ACC1*), and *ACACB* (also known as *ACC2*), acetyl-coenzyme A synthetase *ACSS3*, fatty acid synthase *FASN*, phospholipid-transporting ATPase *ABCA1*, bile salt export pump *ABCB11,* and inositol polyphosphate 4-phosphatase INPP4B (Figure 5). Interestingly, mutations in the five genes - *CUBN, ACACA, ACACB, FASN,* and* PLB1* among the top ten mutations were present in lipid and vitamin cofactor super-pathways (Figure 5), indicating that these two processes might be crucial to the development of ESCC.

A subset of genes harbored a relatively high frequency of mutations and was affected by copy number gain in these super-pathways. These genes included *UROC1* (amino acid super-pathway), *SI* (carbohydrate super-pathway), *CACNA1E, CACNA1C, ADCY2, ADCY8, ADCY1, CACNA1D, ITPR2* (energy super-pathway),* PIK3CA* (lipid super-pathway), *DPYS* (nucleotide super-pathway), *NDUFS8,* and* SDHA* (TCA cycle), and *ALDH1L1* (vitamin cofactor super-pathway) (Figure 5). Unexpectedly, we found several genes, such as* SLC2A2* (also known as *GLUT2*), having comparable rates of gain and loss in different patients. These findings suggested that different tumors might have adopted different metabolic dependencies.

Besides the seven major metabolic super-pathways, we also identified other metabolic processes significantly altered in ESCC in a non-prior-defined view. The Gene Ontology (GO) enrichment analysis of the top 23 highly frequently mutated genes (non-silent mutation frequency>3%) suggested that pathways on circadian entrainment, calcium signaling, ABC transporters, cAMP signaling, insulin secretion, carbohydrate digestion, and absorption, and oxytocin signaling were significantly enriched (Figure S5A). In addition, high mutation frequencies in circadian entrainment pathway genes further highlight a close relationship between circadian rhythm and metabolism in ESCC.

In addition, we examined somatic CNAs, especially those CNAs with a frequency of over 35%. KEGG pathway enrichment analysis of these genes revealed crucial roles of glycosaminoglycan degradation, insulin secretion, phosphatidylinositol signaling, TCA cycle, carbohydrate digestion and absorption, purine metabolism, and regulation of lipolysis in adipocytes in the development of ESCC (Figure S5B). Furthermore, we analyzed the genes with a high frequency of mutation rate (over 35%) or copy number alteration rate (over 35%). These findings further confirmed the significance of pathways related to circadian entrainment, calcium signaling, glycosaminoglycan degradation, insulin secretion, carbohydrate digestion and absorption, oxytocin signaling, and ABC transporters in ESCC and identified pathways related to galactose metabolism and central carbon metabolism in ESCC (Figure S5C). Furthermore, we observed that glutamatergic, cholinergic, and GABAergic synapses were also significantly enriched by mutations and CNA-affected genes (Figure S5A-C), indicating that non-neuronal/neuronal synapse systems might promote tumorigenesis of ESCC.

### Identification of genes associated with pathological progression of ESCC

Tumors with different pathological statuses may have different metabolic adaptations. To determine pathological progression associated with metabolic genes in ESCC, we analyzed the correlation between gene alteration and different clinical outcomes, including tumor stage, grade, and lymph node metastasis (LNM). We observed a significant positive correlation between the three mutated genes and the tumor stage; thirty-five mutated genes positively correlated with the tumor grade, and seven mutated genes positively correlated with LNM (0.05, Fisher's exact test. Table S10). However, no overlapping was observed among the genes. We found that 6, 99, and 81 metabolic genes positively correlated with the tumor stage, tumor grade, and LNM, respectively, for CNA gain-affected genes. Similarly, 23, 282, and 35 metabolic genes correlated with the tumor stage, tumor grade, and LNM, respectively, for CNA loss affected genes (0.05, Fisher's exact test. Table S11, S12). As per GO enrichment analysis, insulin signaling pathway, pyrimidine metabolism, GPI anchor biosynthesis, oxidative phosphorylation, and fatty acid metabolism are pathways significantly affected by these ESCC progression-associated genes (Figure S6A-C).

**Figure 6:**
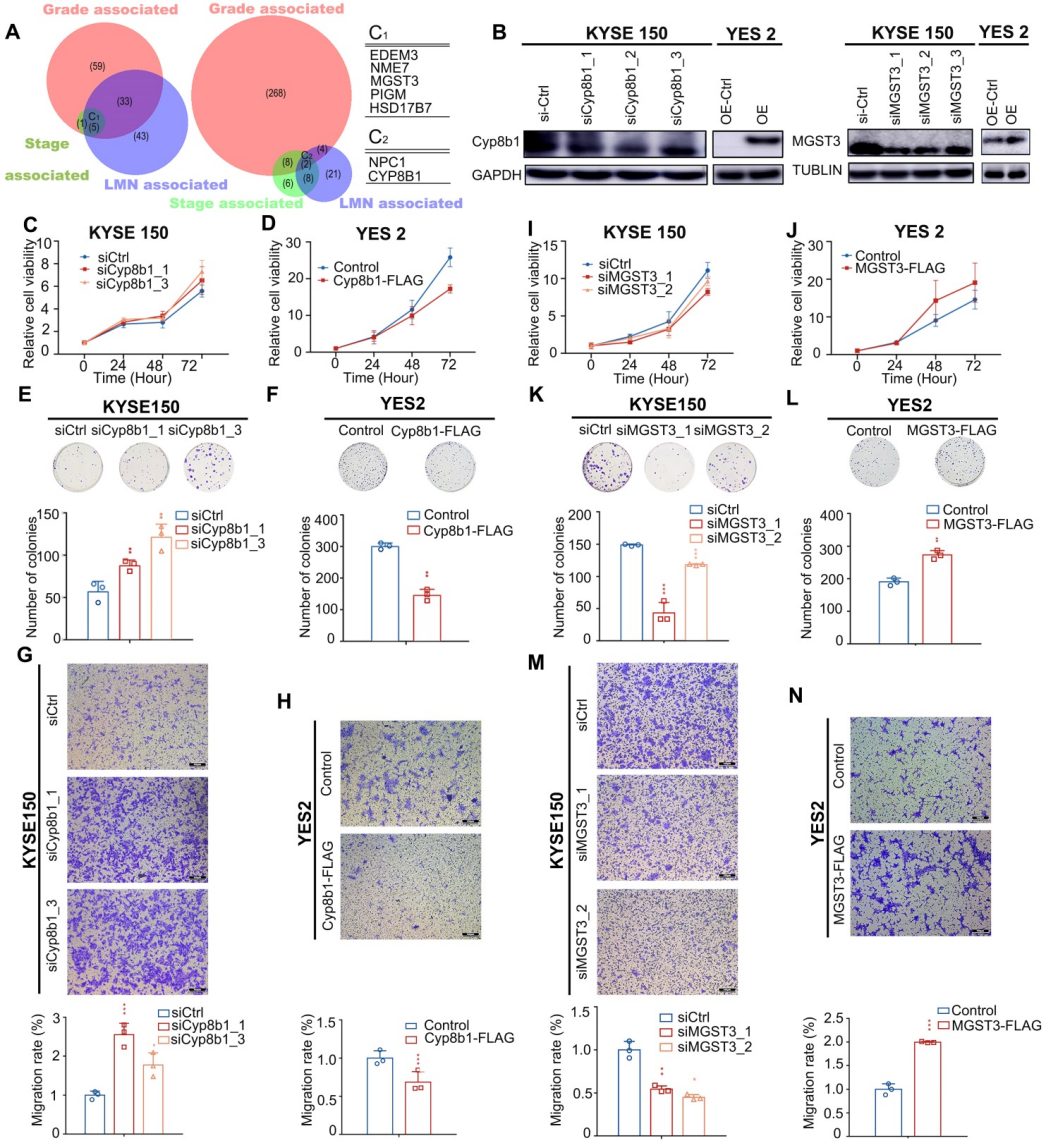
** Pathological progression association analysis of metabolic genes of ESCC reveals potential metabolic driver genes in ESCC. (A)** Venn diagram of CNA-gain genes (left) and CNA-loss genes (right) associated with stage, grade, and LMN. C1 and C2 represent overlapped genes. Copy number alteration of these genes (C1 and C2) are associated with stage, grade, and LMN (0.05). **(B)** Left: Western blot analysis of the protein level of Cyp8b1 in KYSE150 cells transfected with si*CYP8B1* or siCtrl, and in *CYP8B1* overexpression cells or control cells (YES2). Right: Western blot analysis of the protein level of MGST3 in KYSE150 transfected with si*MGST3* or siCtrl, and in *MGST3* overexpression cells and control cells (YES2). **(C-H)** Knockdown of *CYP8B1* enhanced cell proliferation, colony formation, and migration. Ectopic expression of *CYP8B1* reduced cell proliferation, colony formation, and migration. **(I-N)** Knockdown of *MGST3* reduced cell proliferation, colony formation, and migration whereas ectopic expression of *MGST3* enhanced cell proliferation, colony formation, and migration. Representative images (top) and quantification (bottom) are shown in colony formation and transwell assays. All experiments were performed at least three times and data were statistically analyzed by a two-sided t-test. * 0.05,** 0.01, *** 0.001 versus control.

By overlapping these three categories, we determined that CNA gain affected the metabolic genes - *NME7* (19.43%),* MGST3* (18.15%),* PIGM* (21.02%),* HSD17B7* (19.43%), and* EDEM3* (18.79%);* CNA* loss affected the metabolic genes - *CYP8B1* (33.12%) and* NPC1* (15.29%). All these genes significantly correlated with tumor stage, tumor grade, and LNM (Figure 6A), highlighting their potential metabolic driver roles in ESCC development. Moreover, the expression levels of these seven genes significantly correlated with their DNA status in the TCGA ESCC cohort (all 0.001, Pearson's correlation coefficients >0.3, Table S9), further substantiating their crucial roles in ESCC.

All of these genes have not been linked to ESCC, therefore, to further determined whether they served as metabolic drivers in ESCC, we selected *MGST3* and *CYP8B1* for further biological investigations to verify our assumption. *MGST3* (microsomal glutathione S-transferase 3), a member of the glutathione S-transferase family, is implicated in various cancers 42, 43. *CYP8B1* encodes a member of the cytochrome P450 superfamily of enzymes involved in bile acid biosynthesis. CNAs often affect the expression of genes. Therefore, to further confirm that they indeed change at protein level consistent with the genome level, we performed IHC in tissue arrays to examine the protein levels of MGST3 and CYP8B1 in ESCC tumor tissues and adjacent normal tissues. The results showed that MGST1 had higher expression and CYP8B1 had lower expression compared with the adjacent tissue (Figure S7). Additionally, we also obtained MGST3 protein expression levels and corresponding patient clinical information from the Proteomics Cohort 34. As expected and consistent with our results, patients with high MGST3 expression levels had a poorer survival prognosis (p = 4.00E-05, Figure S8B). Due to strict filtering criteria (proteins that were quantified with high confidence in at least half samples), CYP8B1 was not included in the dataset of this cohort, possibly because CYP8B1 was a copy number loss-related gene and was not expressed in all tissues, so it was filtered out, which was in line with expectations. Collectively, these data indicated that MGST3 and CYP8B1 were dysregulated in ESCC and may play important roles in the progression of ESCC.

Intriguingly, the overexpression of *MGST3* significantly promoted while knockdown of *MGST3* attenuated the cell proliferation, colony formation, and cell migration potential of ESCC cell lines - YES2 and KYSE150 (Figure 6B, I-N). Similarly, knockdown of *CYP8B1* significantly enhanced while overexpression of *CYP8B1* reduced the cell proliferation, colony formation, and cell migration potential of ESCC cell lines - YES2 and KYSE150 (Figure 6B-H). Taken together, these findings supported our notion that MGST3 and CYP8B1 play essential roles in the progression of ESCC.

### Clinical implications of altered metabolic genes revealed independent prognostic indicators

At last, we aimed to identify effectively prognostic indicators from metabolic genes. For this purpose, we developed rigorous criteria to screen potential candidates: (i) genes that mutated in at least six patients, (ii) genes with CNAs (gain or loss) occurring in at least 25% of the patients, (iii) both univariate Cox hazard analysis and multivariate Cox hazard analysis (adjusted by age, gender, LNM, drinking, and smoking) reporting a significantly statistical significance.

**Figure 7:**
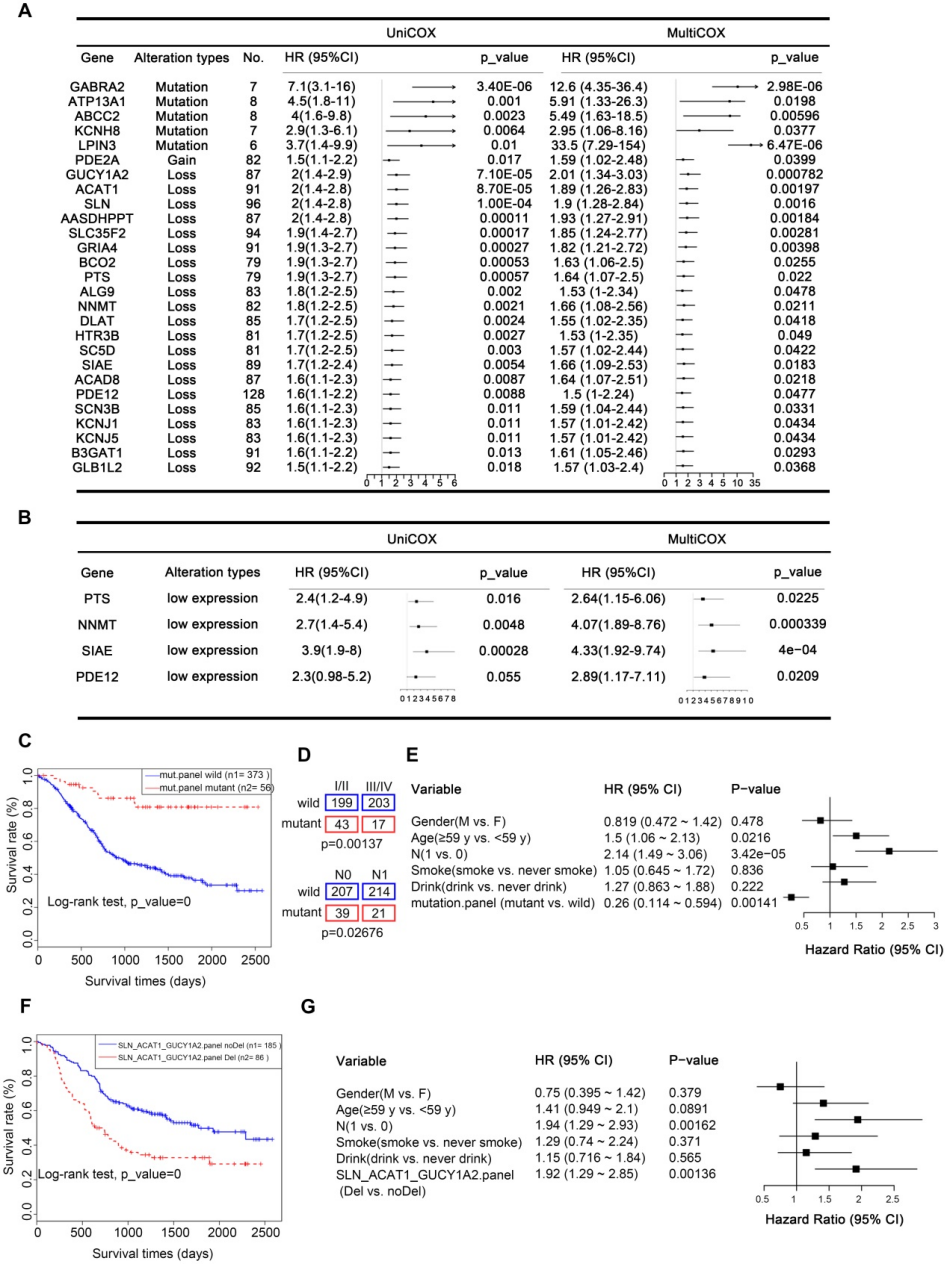
** Prognostic implications of altered metabolic genes. (A)** Twenty-seven metabolic genes with indicated genomic alterations could serve as independent prognostic factors for outcome in ESCC. **(B)** Validated prognostic markers in the independent Proteomics Cohort. **(C)** Kaplan-Meier survival curve for the mutated metabolic gene panel (*ADCY2, CACNA1D, GRIK2, KCNMA1, KCNQ3*). **(D)** The mutated metabolic gene panel was associated with the stage (top) and lymph node metastasis (bottom). **(E)** Multivariate Cox regression survival analysis of the mutated metabolic gene panel. **(F)** Kaplan-Meier survival curve for the CNA-loss affected metabolic gene panel (*SLN, ACAT1,* and *GUCY1A2*). **(G)** Multivariate Cox regression survival analysis of the CNA-loss affected metabolic gene panel.

Our analysis uncovered 27 metabolic genes with genomic alterations meeting the above criteria, which could serve as independent prognostic factors for ESCC (Figure 7A). These genes included five mutated genes (*GABRA2, ATP13A1, ABCC2, KCNH8, LPIN3*), one CNA gain affected gene (*PDE2A*), and twenty-one CNA loss affected genes (*GUCY1A2, ACAT1, SLN, AASDHPPT, SLC35F2, GRIA4, BCO2, PTS, ALG9, NNMT, DLAT, HTR3B, SC5D, SIAE, ACAD8, PDE12, SCN3B, KCNJ1, KCNJ5, B3GAT1,* and* GLB1L2*).

From another perspective, changes at the gene level are ultimately reflected in differences at the protein level. CNAs often directly affect gene expression, which is ultimately reflected in differential levels of proteins. Therefore, to provide more biologically meaningful clinical predictive molecular markers, we validated our prognostic markers in the Proteomics Cohort 34. As mentioned above, due to strict filtering criteria, there were about half of CNAs-affected genes (13/22) were found in this dataset, genes excluded possibly because they were copy number loss-related genes and were not expressed in all tissues. The results identified that there were 4 out of 13 of these proteins, including PTS, NNMT, SIAE, and PDE12 were significantly associated with disease-free survival (DFS), which further confirmed their prognostic value in clinical practice (Figure 7B, Table S13). However, there was just one protein SLC35F2 significantly associated with overall survival (OS, Supplementary Figure S8). This inconsistency may be because overall survival is affected by factors other than the disease itself. These findings further confirmed the prognostic value of these genes in clinical practice. Another possible reason is that the data of the Proteomics Cohort we downloaded from the published article for verification is normalized and transformed, which reflects the relative expression level of the protein, but not the quantified level of the protein, weakening the correlation of protein expression with OS.

A panel of genes may be better than a single gene to indicate prognosis. To achieve robust prognostic prediction, we applied Lasso regression analysis to find the rational panels. By including all the mutated genes, the Lasso regression model yielded a panel with five metabolic genes (*ADCY2, CACNA1D, GRIK2, KCNMA1,* and* KCNQ3*), using which we effectively separated the patients with the better prognostic outcome (with the mutated panel, 56) from all patients (0.0001, Kaplan-Meier survival analysis and Log-rank test, Figure 7C). Statistical analyses revealed that patients with or without this panel of mutated genes were significantly negatively correlated with tumor stage (p = 0.00137) and lymph node metastasis (LNM, p = 0.02676, Figure 7D). The multivariate Cox regression survival analysis adjusted for age, gender, LNM, and drinking and smoking status demonstrated a strong correlation between the presence of this mutated panel and overall survival (p = 0.00141, HR = 0.26, 95% CI 0.114 to 0.594, Figure 7E). These findings indicate that this mutated panel is an independent prognostic factor for ESCC. The stratification by the mutated panel displayed even higher prognostic significance than the widely employed LNM (p = 3.42e-05, HR = 2.14 95% CI 1.49 to 3.06, Figure 7E). Nonetheless, we should note that due to the limited number of mutations, these results are only suggestive and more experimental validation is required.

Additionally, the predictive value of each gene was listed in Figure S9A-E and most of them displayed a significant association prognosis. Specifically,* GRIK2* was mutated in 13 of 490 (2.65%) patients in our integrated ESCC cohort. *GRIK2* mutations were associated with a better prognosis (p = 0 .024) (Figure S9A). *GRIK2* belongs to the kainate family of glutamate receptors. Mutations in this gene are associated with autosomal recessive cognitive disability 44. Almost half of the mutations occur in the ANF receptor domain, which is different from prevalent mutations in neurodevelopmental deficits disease 45. Previous studies have shown that *GRIK2* may play a tumor-suppressor role in gastric cancer. Mutations in* ADCY2* also led to a better prognosis (p = 0 .04) (Figure S9B) 46. *ADCY2* is associated with colorectal cancer metastasis 47. In our study, patients with *KCNMA1* mutations (11, 2.24%) also proved to have a longer survival time (p = 0 .031) (Figure S9C). As a critical tumor suppressor gene involved in gastric carcinogenesis 48, *KCNMA1* has not been extensively studied in ESCC. Surprisingly, three of the mutations were in-frame deletions (c.(133-138) tcctct>tct)). These findings point toward a new function of *KCNMA1* in ESCC (Figure S9J).

Similarly, Lasso regression analysis also revealed that a panel composed of three CNA loss-affected metabolic genes (*SLN, ACAT1,* and *GUCY1A2*) is associated with poor prognosis in patients with ESCC (0.0001, Kaplan-Meier survival analysis and Log-rank test, Figure 7F), also confirmed to be an independent prognostic factor by multivariate Cox regression survival analysis (p = 0.00136, HR = 1.92, 1.29 to 2.85, adjusted for age, gender, LNM, and drinking and smoking status, Figure 7G). The predictive value of each gene was also listed in Figure S9 and all of them displayed a significant association prognosis (Figure S9F-H). From another perspective, CNAs often directly affect gene expression. Therefore, to provide more biologically meaningful clinical predictive molecular markers, we validated our CNA prognostic panel in the TCGA mRNA Cohort (p = 0.034, Figure S9I). Consistently, patients with low expression of any of these CNA genes had a poorer prognosis.

## Discussion

In this study, we performed a meta-analysis of seven previously published ESCC cohort studies about genomic metabolic alterations. Analysis of gene alterations and pathway enrichment from 490 ESCC samples revealed a diverse metabolic landscape, giving us a comprehensive understanding of ESCC metabolic reprogramming and potential biomarkers and therapeutic targets of ESCC. Our analysis uncovered a panel of metabolic genes harboring highly frequent mutations or significantly affected by CNAs and metabolic genes with recurrently mutated residues, providing the genomic basis for the significant involvement of metabolism in ESCC tumorigenesis. Intriguingly, patients with ESCC had remarkable metabolic heterogeneity in mutation profiles, CNA patterns, and metabolic pathways. Clinical studies revealed that a subset of metabolic genes are significantly associated with pathological progression of ESCC (stage, grade, and lymph node metastasis) and reported 27 metabolic genes with genomic alterations, which could serve as independent prognostic factors. Most importantly, using molecular subtyping by CNA clusters, mutation panel (*ADCY2, CACNA1D, GRIK2, KCNMA1,* and* KCNQ3*), and genes with CNA loss panel (*SLN, ACAT1, GUCY1A2*), we stratified ESCC patients into distinct prognostic subgroups. *In vitro* investigations confirmed that the mutations in *RYR2, MGST3,* and* CYP8B1* functionally contribute to the development of malignant phenotypes of ESCC*.* The present study represents a comprehensive characterization of metabolic genomic alterations in ESCC. Further studies of the biological and therapeutic significance of the potential metabolic drivers are expected to lead to the development of effective diagnostic and therapeutic approaches for ESCC.

In view of mutation frequency and recurrent residues, our analysis highlighted significant alterations in metal ion channels/transporters - including calcium, potassium, and sodium ion transporters. Metal ion channels are membrane proteins that integrate extracellular and intracellular responses *via* transporting ions. Since these ion channels contribute to all hallmarks of cancer, Natalia Prevarskaya and colleagues proposed that cancers may be classified as special types of oncochannelopathies 49. Furthermore, recent studies have extended our understanding that dysregulation of metal ion (Ca^2+^/K^+^/Na^+^) homeostasis could remodel the tumor microenvironment and impair the functions of tumor-infiltrating lymphocytes 49-53. Therefore, the alterations in metal ion channels may benefit the ESCC immunotherapy.

We observed that seven major metabolic super-pathways - amino acid metabolism, carbohydrate metabolism, integration of energy, lipid metabolism, nucleotide metabolism, tricarboxylic acid cycle (TCA cycle), and vitamin and cofactor metabolism - are significantly affected in ESCC, demonstrating highly heterogeneous mutational profiles and similarities in CNA alteration frequencies. Our analysis identified significant variations in the lipid super-pathway, especially in the metabolic process of the central metabolite and second messenger - acetyl-coenzyme A (acetyl-CoA) 54. We also found highly frequent mutations in carboxylase ACACA (also known as ACC1), ACACB (also known as ACC2), and acetyl-coenzyme A synthetase ACSS3. Besides, we found that genes - *PTEN, RPE65, CUBN, CACNA1H, ABCG1,* and *DHRS4* - participating in alcohol metabolism are frequently mutated. The presence of these mutations may be associated with the risk of predisposition to alcohol consumption. Since the glutamatergic, cholinergic, and GABAergic synaptic pathways were significantly enriched by mutations and CNA-affected genes in our integrated ESCC cohort, we believe that the non-neuronal synapse systems might be dysregulated during the development of ESCC. Previous studies also revealed that the non-neuronal synapse systems play crucial roles in epithelial cell malignancy, including breast cancer 55, lung cancer 56, melanoma, and renal cell carcinoma 57. Our findings further supported the notion that non-neuronal synapse systems dysregulations contribute to tumorigenesis. Recently, several studies have found that tumor cells can form pseudo-tripartite synapses with neurons to increase tumor growth and promote brain metastasis 58-60. Thus, the alterations of glutamatergic, cholinergic, and GABAergic synapses pathways may functionally regulate ESCC cell malignant phenotypes *via* non-neuronal and neuronal signaling. Our further understanding of the nervous system's role in tumor development will highlight new therapeutic avenues for ESCC; targeting neuron-cancer cell interactions may prove to be a promising therapeutic strategy 61.

Several researchers have developed various algorithms to identify tumor driver genes 62, 63; however, only a few significant candidates were associated with ESCC 64. Somatic mutation drivers only partially explain the biological complexity of tumors. Relying on such mutation drivers may hamper the development of novel diagnostic markers and therapeutic targets. A pan-cancer analysis of passenger mutations in more than 2,500 cancer genomes revealed other than standard drivers, passengers also play roles in tumorigenesis 65. Therefore, in this study, we developed rigorous criteria to identify potential metabolic drivers associated with ESCC: alterations (mutation and CNAs) frequency, genes with recurrent residues, and genes associated with pathological progressions (stage, grade, lymph node metastasis, and prognosis). Moreover, we also performed *in vitro* experiments to validate the potential candidates driving ESCC progression. We found 12 highly frequent mutated genes (*RYR2, PIK3CA, ABCA13, SI, RYR3, MGAM, CUBN, HCN1, CACNA1H, ADCY2, ABCA4, PTEN,* and *ADCY8*), and a subset of genes represented a phenomenon of recurrent residue enrichment, such as *ACOXL* (p.A578S, 4/6), *ST6GALNAC5* (p.Q49del, 3/6), *ABCG1* (p.A203E, 3/5), *PFKFB2* (p.R92W, 2/4). Regarding the relationship between metabolic alterations and pathological features, our data revealed 7 genes significantly positive associated with stage, grade, and LNM, highlighting their roles in ESCC development.

Among these potential metabolic drivers, *RYR2*, which encodes a large protein, was the most frequently mutated gene. Although generally considered a passenger, *RYR2* was found to have low expression in tumor tissues. *RYR2* is associated with poor prognosis in head and neck cancers and thyroid carcinoma 66, 67. We also found a lower expression level of *RYR2* in tumor tissues than in the adjacent healthy counterparts. *RYR2* was also associated with a poor prognosis. Interestingly, we also observed that patients positive for cytoplasmic and nuclear positive staining of *RYR2* had better overall survival than those positive for cytoplasmic staining. Our study uncovered a novel mechanism in which *RYR2* competitively interacted with the E3 ubiquitin ligase and MDM2 and protected p53 from MDM2-mediated degradation; however, *RYR2* was frequently downregulated in ESCC tumor tissues, which led to decreased protein levels of p53 and p21, promoting malignancy in ESCC. These findings suggest that though not predicted to be a driver, metabolic genes with a high frequency of mutation may also contribute to tumorigenesis.

We further confirmed the notion that alterations in metabolic genes positively correlated with pathological progression may function as potential drivers. The deletion of *CYP8B1* and amplification of *MGST3* were simultaneously associated with tumor grade, stage, and LNM in ESCC. CYP8B1 participates in bile acid synthesis and acts as a prognosis‑associated biomarker for hepatocellular carcinoma (HCC) 68. MGST3 is involved in leukotrienes and prostaglandin E production and acts as a critical oxidative stress-related gene 43. This study found that overexpression of MGST3 or knocking down CYP8B1 improves cell proliferation, colony formation, and cell migration.

It was important to emphasize that here we also provided the genomic perspective of prognosis-relevant metabolic genes. We found that 27 metabolic genes that harbored mutations or are affected by CNAs could serve as independent prognostic factors. Interestingly, patients with mutations in GABA-A receptor (GABRA2), which mediates the transport of major inhibitory neurotransmitter GABA, had a high risk of poor outcomes. GRIA4 (Glutamate Ionotropic Receptor AMPA Type Subunit 4) is a predominant excitatory neurotransmitter receptor; patients with CNA loss of *GRIA4* had poor survival outcomes. These findings further highlighted the importance of synaptic system dysregulation in ESCC development. We developed two prognostic classifier panels that could stratify patients with ESCC into two distinct survival outcome groups.

Although the present study is the most comprehensive in terms of metabolic genomic alterations and has the largest sample size of ESCC patients, there are several limitations. Firstly, the molecular subtyping by CNA patterns lacked independent validations, and we did not study the correlations between the three clusters and the previously known ESCC drivers. Secondly, although we identified recurrent mutations in several genes, the same was not functionally examined. Finally, the mutation prognostic panel and the CNA loss prognostic panel were not validated in an independent cohort, limiting their applicability in clinical practice.

In summary, we characterized the genomic basis of metabolic alterations in the largest integrated ESCC cohort. We found that mutations and copy number variations in metabolic genes occurred across all samples; however, the mutations, CNAs events, and the alterations in metabolic pathways were highly heterogeneous in different samples. We identified a subset of potential metabolic drivers in ESCC using alteration frequencies, recurrent residues, pathological progression associations, and biological validations. Our study provided deep insights into the metabolic landscape in ESCC, which extended our understanding of the genomic basis of metabolic reconfiguration. In addition, our findings highlighted the prognostic utility of the genes and identified potential therapeutic targets for further validation.

## Materials and methods

### Sample and genome data collection and clinical features of ESCC patients

We collected genome data of 490 paired ESCC samples from seven publications, which have been analyzed by our previous study 28. The clinical data were also acquired.

### Mutational signature analysis

Mutational signatures of metabolic genes associated with ESCC were displayed using a 96-context classification. Nonnegative matrix factorization (NMF) was used to identify the operative processes based on the reproducibility of the signatures and low error for reconstructing the original catalogs 69-71. We then performed hierarchical clustering based on the enrichment of specific mutational signatures.

### Copy number alterations analysis

Copy number alterations analysis was performed as described 28. Briefly, copy number alterations (CNAs) were detected with SegSeq for 31 WGS, and GATK4 Alpha for 283 WES. Hierarchical clustering analysis on the CNA of metabolic-related genes revealed three clusters within the study patients.

### Cell cultures

The human esophageal squamous cell carcinoma cell lines YES2 and KYSE150 were kindly provided by Dr. Y. Shimada (Kyoto University). These cells were cultured in RPMI 1640 (Lonza, Switzerland) supplemented with 8% fetal bovine serum (Gibco, America) and 1% penicillin/streptomycin (Gibco, America). Both types of ESCC cells were maintained at 37 °C and 5% CO_2_.

### Antibodies

The antibodies used included Ryanodine Receptor (Thermo Fisher, MA3-916), and CYP8B1 (Thermo Fisher, PA5-37088), all of which were purchased from Thermo Fisher (America. p53 (ab131442), IgG R (ab172730), MGST3 (ab192254) were purchased from Abcam (United Kingdom). MDM2 (#86934s), ubiquitin (#3933s), α/β-Tubulin (#2148) were purchased from Cell Signaling. GAPDH (P01L01) was purchased from Gene-Protein Link.

### Cell proliferation assay

The proliferation ability of cells was detected by MTS assays (Promega) according to the manufacturer's instructions. The cells in the logarithmic growth phase were digested with 0.25% trypsin (Gibco, America), the cells were resuscitated in a complete culture medium, the cells were counted and the density was adjusted, the cells were inoculated in a 96-well plate with 1 × 10^4^ cells per well, and blank control was set up. After 24, 48, and 72 h, 10 μL (15 mg/mL) MTS reagent was added to each well and cultured in the incubator for 2 h, and the absorbance was detected. The data were analyzed with GraphPad Prism 5 software and are presented as the percentage (%) of cell viability relative to the control.

### Colony formation assay

The cells in the logarithmic growth phase were digested with 0.25% trypsin and resuscitated in the complete culture medium. The cells were counted and the density was adjusted. The cells were inoculated in 6-well plates with 2 × 10^3^ cells per well and cultured for 14 days. After 14 days, the cultures were washed with PBS, fixed with methanol for 15 min, and stained with a 0.1% crystal violet solution for 30 min. Then the culture was washed with deionized water, the water was drained and photographed under a microscope.

### Transwell assay

For transwell assay, the cells in the logarithmic growth phase were digested with 0.25% trypsin and suspended in a serum‐free culture medium. The cells were counted and the density was adjusted. The cells were placed on the top of the up chambers (Corning), and a 600 μl culture medium containing 20% fetal bovine serum was added to the lower chamber as a chemical attractant. After 24 h, discard the upper chamber culture medium, wash gently with PBS, fixed with 0.5% crystal violet solution at room temperature for 10 min, wash with deionized water, remove the excess dye, drain the water and take pictures under the microscope.

### Western blot

After treatment with small interfering RNA (siRNA)/plasmid for 48 h, cells were harvested in RIPA buffer (Beyotime, China). A total of 20 μg of cellular protein was subjected to 10%-15% SDS-polyacrylamide gel electrophoresis and transferred onto a polyvinylidene difluoride membrane. Incubation with antibodies was performed as described previously. The chemiluminescence signals were detected with an Amersham Imager 600 (GE, America).

### Immunoprecipitation (IP) assay

For immunoprecipitation, KYSE30 and YES2 cells were lysed with lysis buffer containing protease inhibitor cocktail (Roche). Protein A/G Magnetic Beads were incubated with control IgG or anti‐RYR2 antibody at 4 °C for 1 h. Then cell extracts were added and incubated at 4 °C for 12 h. After washing, precipitated proteins were detected by Western blot with an antibody against MDM2.

### Quantitative Immunoprecipitation (IP) assay

After treatment with small interfering RNA (siRNA) for 48 h, KYSE150 and YES2 cells were lysed with lysis buffer containing protease inhibitor cocktail (Roche), and protein was quantized by BCA assay. Magnetic beads were incubated with control IgG or anti-p53 antibody at 4 °C for 1 h. Then an equal amount of cell extracts was added and incubated at 4 °C for 12 h. After washing, precipitated proteins were detected by Western blot with an antibody against MDM2.

### Ubiquitination Immunoprecipitation assay

After treatment with small interfering RNA (siRNA) for 42 h, KYSE150 and YES2 cells were treated with 10 μM MG132 for 6 h to inhibit proteasome degradation. Then, treated cells were lysed with lysis buffer containing protease inhibitor cocktail. Magnetic beads were incubated with control IgG or anti‐p53 antibody at 4 °C for 1 h. Then cell extracts were added and incubated at 4 °C for 12 h. After washing, precipitated proteins were detected by Western blot with an antibody against ubiquitin.

### Immunohistochemistry

Immunohistochemistry (IHC) analysis was performed as described before 72. In brief, the sections were deparaffinized with xylenes and rehydrated in graded ethanol. Sections were submerged into EDTA antigenic retrieval buffer (pH8.0) and microwaved for antigenic retrieval. The sections were then treated with 3% hydrogen peroxide in methanol to quench the endogenous peroxidase activity, followed by incubation with 1% goat serum albumin to block nonspecific binding. The tissue sections were incubated with rabbit anti-RYR2 (1:200; Thermo Fisher, MA3-916), rabbit anti-CYP8B1 (1:200; Thermo Fisher, PA5-37088), rabbit anti-MGST3 (1:200; Abcam, ab192254), overnight at 4 °C. After washing, the tissue sections were treated with goat anti-mouse/rabbit IgG HRP-polymer (ZSGB-BIO, Beijing, China) for 20 min. 3, 3'-Diaminobenzidine was used as the chromogen.

The IHC score was determined by multiplying the score of staining intensity by the score of positive area. First, the intensity was graded as follows: 0, negative; 1, weak; 2, moderate; 3, strong. Second, the proportion of positive tumor cells was graded: 0, <5%; 1, 5-25%; 2, 26-50%; 3, 51-75%; 4, >75%. A final score was derived by the multiplication of these two primary scores. Final scores of 0-3 were defined as 'low expression' (-); scores of 4-8 as 'high expression' (+). Additionally, patients were divided into two groups based on RYR2 whether positive staining in nuclear or not.

## Supplementary Material

Supplementary figures.

Supplementary tables.

## References

[1] Hanahan D, Weinberg RA (2011). Hallmarks of cancer: the next generation. Cell.

[2] Warburg O, Wind F, Negelein E (1927). THE METABOLISM OF TUMORS IN THE BODY. J Gen Physiol.

[3] Oermann EK, Wu J, Guan KL, Xiong Y (2012). Alterations of metabolic genes and metabolites in cancer. Semin Cell Dev Biol.

[4] Pavlova NN, Thompson CB (2016). The Emerging Hallmarks of Cancer Metabolism. Cell Metab.

[5] Hu J, Locasale JW, Bielas JH, O'Sullivan J, Sheahan K, Cantley LC, Vander Heiden MG (2013). Heterogeneity of tumor-induced gene expression changes in the human metabolic network. Nat Biotechnol.

[6] Nilsson R, Jain M, Madhusudhan N, Sheppard NG, Strittmatter L, Kampf C (2014). Metabolic enzyme expression highlights a key role for MTHFD2 and the mitochondrial folate pathway in cancer. Nat Commun.

[7] Possemato R, Marks KM, Shaul YD, Pacold ME, Kim D, Birsoy K (2011). Functional genomics reveal that the serine synthesis pathway is essential in breast cancer. Nature.

[8] Amary MF, Damato S, Halai D, Eskandarpour M, Berisha F, Bonar F (2011). Ollier disease and Maffucci syndrome are caused by somatic mosaic mutations of IDH1 and IDH2. Nat Genet.

[9] Mardis ER, Ding L, Dooling DJ, Larson DE, McLellan MD, Chen K (2009). Recurring mutations found by sequencing an acute myeloid leukemia genome. N Engl J Med.

[10] Chen C, Liu Y, Lu C, Cross JR, Morris JP 4th, Shroff AS (2013). Cancer-associated IDH2 mutants drive an acute myeloid leukemia that is susceptible to Brd4 inhibition. Genes Dev.

[11] Dang L, White DW, Gross S, Bennett BD, Bittinger MA, Driggers EM (2009). Cancer-associated IDH1 mutations produce 2-hydroxyglutarate. Nature.

[12] Lu C, Venneti S, Akalin A, Fang F, Ward PS, Dematteo RG (2013). Induction of sarcomas by mutant IDH2. Genes Dev.

[13] Reitman ZJ, Duncan CG, Poteet E, Winters A, Yan LJ, Gooden DM (2014). Cancer-associated isocitrate dehydrogenase 1 (IDH1) R132H mutation and d-2-hydroxyglutarate stimulate glutamine metabolism under hypoxia. J Biol Chem.

[14] Baysal BE, Ferrell RE, Willett-Brozick JE, Lawrence EC, Myssiorek D, Bosch A (2000). Mutations in SDHD, a mitochondrial complex II gene, in hereditary paraganglioma. Science.

[15] Niemann S, Müller U (2000). Mutations in SDHC cause autosomal dominant paraganglioma, type 3. Nat Genet.

[16] Astuti D, Douglas F, Lennard TW, Aligianis IA, Woodward ER, Evans DG (2001). Germline SDHD mutation in familial phaeochromocytoma. Lancet.

[17] Hao HX, Khalimonchuk O, Schraders M, Dephoure N, Bayley JP, Kunst H (2009). SDH5, a gene required for flavination of succinate dehydrogenase, is mutated in paraganglioma. Science.

[18] Bayley J.-P, P (2005). Devilee, and P.E.J.B.m.g. Taschner, The SDH mutation database: an online resource for succinate dehydrogenase sequence variants involved in pheochromocytoma, paraganglioma and mitochondrial complex II deficiency. BMC Med Genet.

[19] Burnichon N, Brière JJ, Libé R, Vescovo L, Rivière J, Tissier F (2010). SDHA is a tumor suppressor gene causing paraganglioma. Hum Mol Genet.

[20] Pantaleo MA, Astolfi A, Indio V, Moore R, Thiessen N, Heinrich MC (2011). SDHA loss-of-function mutations in KIT-PDGFRA wild-type gastrointestinal stromal tumors identified by massively parallel sequencing. J Natl Cancer Inst.

[21] Korpershoek E, Favier J, Gaal J, Burnichon N, van Gessel B, Oudijk L (2011). SDHA immunohistochemistry detects germline SDHA gene mutations in apparently sporadic paragangliomas and pheochromocytomas. J Clin Endocrinol Metab.

[22] Tomlinson IP, Alam NA, Rowan AJ, Barclay E, Jaeger EE, Kelsell D (2002). Germline mutations in FH predispose to dominantly inherited uterine fibroids, skin leiomyomata and papillary renal cell cancer. Nat Genet.

[23] Choi H, Na KJ (2018). Pan-cancer analysis of tumor metabolic landscape associated with genomic alterations. Mol Cancer.

[24] Sinkala M, Mulder N, Patrick Martin D (2019). Metabolic gene alterations impact the clinical aggressiveness and drug responses of 32 human cancers. Commun Biol.

[25] Haider S, McIntyre A, van Stiphout RG, Winchester LM, Wigfield S, Harris AL (2016). Genomic alterations underlie a pan-cancer metabolic shift associated with tumour hypoxia. Genome Biol.

[26] Peng X, Chen Z, Farshidfar F, Xu X, Lorenzi PL, Wang Y (2018). Molecular Characterization and Clinical Relevance of Metabolic Expression Subtypes in Human Cancers. Cell Rep.

[27] Shang L, Wang M (2013). Molecular alterations and clinical relevance in esophageal squamous cell carcinoma. Front Med.

[28] Du P, Huang P, Huang X, Li X, Feng Z, Li F (2017). Comprehensive genomic analysis of Oesophageal Squamous Cell Carcinoma reveals clinical relevance. Sci Rep.

[29] Phan NN, Wang CY, Chen CF, Sun Z, Lai MD, Lin YC (2017). Voltage-gated calcium channels: Novel targets for cancer therapy. Oncol Lett.

[30] Khammanivong A, Anandharaj A, Qian X, Song JM, Upadhyaya P, Balbo S (2016). Transcriptome profiling in oral cavity and esophagus tissues from (S)-N'-nitrosonornicotine-treated rats reveals candidate genes involved in human oral cavity and esophageal carcinogenesis. Mol Carcinog.

[31] Zhang JY, Zhang PP, Zhou WP, Yu JY, Yao ZH, Chu JF (2019). L-Type Cav 1.2 Calcium Channel-α-1C Regulates Response to Rituximab in Diffuse Large B-Cell Lymphoma. Clin Cancer Res.

[32] Chang MT, Asthana S, Gao SP, Lee BH, Chapman JS, Kandoth C (2016). Identifying recurrent mutations in cancer reveals widespread lineage diversity and mutational specificity. Nat Biotechnol.

[33] Nath A, Chan C (2016). Genetic alterations in fatty acid transport and metabolism genes are associated with metastatic progression and poor prognosis of human cancers. Sci Rep.

[34] Liu W, Xie L, He YH, Wu ZY, Liu LX, Bai XF (2021). Large-scale and high-resolution mass spectrometry-based proteomics profiling defines molecular subtypes of esophageal cancer for therapeutic targeting. Nat Commun.

[35] Boehme KA, Blattner C (2009). Regulation of p53-insights into a complex process. Crit Rev Biochem Mol Biol.

[36] Waters CE, Saldivar JC, Hosseini SA, Huebner K (2014). The FHIT gene product: tumor suppressor and genome "caretaker". Cell Mol Life Sci.

[37] Kim J, Hu Z, Cai L, Li K, Choi E, Faubert B (2017). CPS1 maintains pyrimidine pools and DNA synthesis in KRAS/LKB1-mutant lung cancer cells. Nature.

[38] Çeliktas M, Tanaka I, Tripathi SC, Fahrmann JF, Aguilar-Bonavides C, Villalobos P (2017). Role of CPS1 in Cell Growth, Metabolism and Prognosis in LKB1-Inactivated Lung Adenocarcinoma. J Natl Cancer Inst.

[39] Yao S, Nguyen TV, Rolfe A, Agrawal AA, Ke J, Peng S (2020). Small Molecule Inhibition of CPS1 Activity through an Allosteric Pocket. Cell Chem Biol.

[40] Taguchi A, Fahrmann JF, Hanash SM (2020). A Promising CPS1 Inhibitor Keeping Ammonia from Fueling Cancer. Cell Chem Biol.

[41] Ma W, Wang S, Zhang T, Zhang EY, Zhou L, Hu C (2018). Activation of choline kinase drives aberrant choline metabolism in esophageal squamous cell carcinomas. J Pharm Biomed Anal.

[42] Zhang J, Xu C, Gao Y, Wang Y, Ding Z, Zhang Y (2020). A Novel Long Non-coding RNA, MSTRG.51053.2 Regulates Cisplatin Resistance by Sponging the miR-432-5p in Non-small Cell Lung Cancer Cells. Front Oncol.

[43] Pedro NF, Biselli JM, Maniglia JV, Santi-Neto D, Pavarino ÉC, Goloni-Bertollo EM (2018). Candidate Biomarkers for Oral Squamous Cell Carcinoma: Differential Expression of Oxidative Stress-Related Genes. Asian Pac J Cancer Prev.

[44] Takenouchi T, Hashida N, Torii C, Kosaki R, Takahashi T, Kosaki K (2014). 1p34.3 deletion involving GRIK3: Further clinical implication of GRIK family glutamate receptors in the pathogenesis of developmental delay. Am J Med Genet A.

[45] Guzmán YF, Ramsey K, Stolz JR, Craig DW, Huentelman MJ, Narayanan V (2017). A gain-of-function mutation in the GRIK2 gene causes neurodevelopmental deficits. Neurol Genet.

[46] Wu CS, Lu YJ, Li HP, Hsueh C, Lu CY, Leu YW (2010). Glutamate receptor, ionotropic, kainate 2 silencing by DNA hypermethylation possesses tumor suppressor function in gastric cancer. Int J Cancer.

[47] Fang LT, Lee S, Choi H, Kim HK, Jew G, Kang HC (2014). Comprehensive genomic analyses of a metastatic colon cancer to the lung by whole exome sequencing and gene expression analysis. Int J Oncol.

[48] Ma G, Liu H, Hua Q, Wang M, Du M, Lin Y (2017). KCNMA1 cooperating with PTK2 is a novel tumor suppressor in gastric cancer and is associated with disease outcome. Mol Cancer.

[49] Prevarskaya N, Skryma R, Shuba Y (2018). Ion Channels in Cancer: Are Cancer Hallmarks Oncochannelopathies?. Physiol Rev.

[50] Sang LJ, Ju HQ, Liu GP, Tian T, Ma GL, Lu YX (2018). LncRNA CamK-A Regulates Ca2+-Signaling-Mediated Tumor Microenvironment Remodeling. Mol Cell.

[51] Newton HS, Gawali VS, Chimote AA, Lehn MA, Palackdharry SM, Hinrichs BH (2020). PD1 blockade enhances K+ channel activity, Ca2+ signaling, and migratory ability in cytotoxic T lymphocytes of patients with head and neck cancer. J Immunother Cancer.

[52] Eil R, Vodnala SK, Clever D, Klebanoff CA, Sukumar M, Pan JH (2016). Ionic immune suppression within the tumour microenvironment limits T cell effector function. Nature.

[53] Vodnala SK, Eil R, Kishton RJ, Sukumar M, Yamamoto TN, Ha NH (2019). T cell stemness and dysfunction in tumors are triggered by a common mechanism. Science.

[54] Pietrocola F, Galluzzi L, Bravo-San Pedro JM, Madeo F, Kroemer G (2015). Acetyl coenzyme A: a central metabolite and second messenger. Cell Metab.

[55] Español AJ, de la Torre E, Fiszman GL, Sales ME (2007). Role of non-neuronal cholinergic system in breast cancer progression. Life Sci.

[56] Saracino L, Zorzetto M, Inghilleri S, Pozzi E, Stella GM (2013). Non-neuronal cholinergic system in airways and lung cancer susceptibility. Transl Lung Cancer Res.

[57] Martino JJ, Wall BA, Mastrantoni E, Wilimczyk BJ, La Cava SN, Degenhardt K (2013). Metabotropic glutamate receptor 1 (Grm1) is an oncogene in epithelial cells. Oncogene.

[58] Venkatesh HS, Morishita W, Geraghty AC, Silverbush D, Gillespie SM, Arzt M (2019). Electrical and synaptic integration of glioma into neural circuits. Nature.

[59] Neman J, Termini J, Wilczynski S, Vaidehi N, Choy C, Kowolik CM (2014). Human breast cancer metastases to the brain display GABAergic properties in the neural niche. Proc Natl Acad Sci U S A.

[60] Zeng Q, Michael IP, Zhang P, Saghafinia S, Knott G, Jiao W (2019). Synaptic proximity enables NMDAR signalling to promote brain metastasis. Nature.

[61] Wang H, Zheng Q, Lu Z, Wang L, Ding L, Xia L (2021). Role of the nervous system in cancers: a review. Cell Death Discov.

[62] Lawrence MS, Stojanov P, Polak P, Kryukov GV, Cibulskis K, Sivachenko A (2013). Mutational heterogeneity in cancer and the search for new cancer-associated genes. Nature.

[63] Dietlein F, Weghorn D, Taylor-Weiner A, Richters A, Reardon B, Liu D (2020). Identification of cancer driver genes based on nucleotide context. Nat Genet.

[64] Cui Y, Chen H, Xi R, Cui H, Zhao Y, Xu E (2020). Whole-genome sequencing of 508 patients identifies key molecular features associated with poor prognosis in esophageal squamous cell carcinoma. Cell Res.

[65] Kumar S, Warrell J, Li S, McGillivray PD, Meyerson W, Salichos L (2020). Passenger Mutations in More Than 2,500 Cancer Genomes: Overall Molecular Functional Impact and Consequences. Cell.

[66] Schmitt K, Molfenter B, Laureano NK, Tawk B, Bieg M, Hostench XP (2019). Somatic mutations and promotor methylation of the ryanodine receptor 2 is a common event in the pathogenesis of head and neck cancer. Int J Cancer.

[67] Xu N, Zhang D, Chen J, He G, Gao L (2019). Low expression of ryanodine receptor 2 is associated with poor prognosis in thyroid carcinoma. Oncol Lett.

[68] Wang X, Liao X, Yang C, Huang K, Yu T, Yu L (2019). Identification of prognostic biomarkers for patients with hepatocellular carcinoma after hepatectomy. Oncol Rep.

[69] Gehring JS, Fischer B, Lawrence M, Huber W (2015). SomaticSignatures: inferring mutational signatures from single-nucleotide variants. Bioinformatics.

[70] Alexandrov LB, Nik-Zainal S, Wedge DC, Campbell PJ, Stratton MR (2013). Deciphering signatures of mutational processes operative in human cancer. Cell Rep.

[71] Alexandrov LB, Kim J, Haradhvala NJ, Huang MN, Tian Ng AW, Wu Y (2020). The repertoire of mutational signatures in human cancer. Nature.

[72] Zhang W, Hong R, Li L, Wang Y, Du P, Ou Y (2018). The chromosome 11q13.3 amplification associated lymph node metastasis is driven by miR-548k through modulating tumor microenvironment. Mol Cancer.

[73] Camp RL, Dolled-Filhart M, Rimm DL (2004). X-tile: a new bio-informatics tool for biomarker assessment and outcome-based cut-point optimization. Clin Cancer Res.

