# Supplementary material for: The metabolic genomic atlas reveals potential drivers and clinically relevant insights into the etiology of esophageal squamous cell carcinoma
Source: Theranostics. 2022 Aug 21;12(14):6160–78. doi: 10.7150/thno.70814 (PMC9475459; doi:10.7150/thno.70814)
Supplement: Supplementary file 1 — Supplementary figures. [file thnov12p6160s1.pdf]

Supplementary Figures and Legends

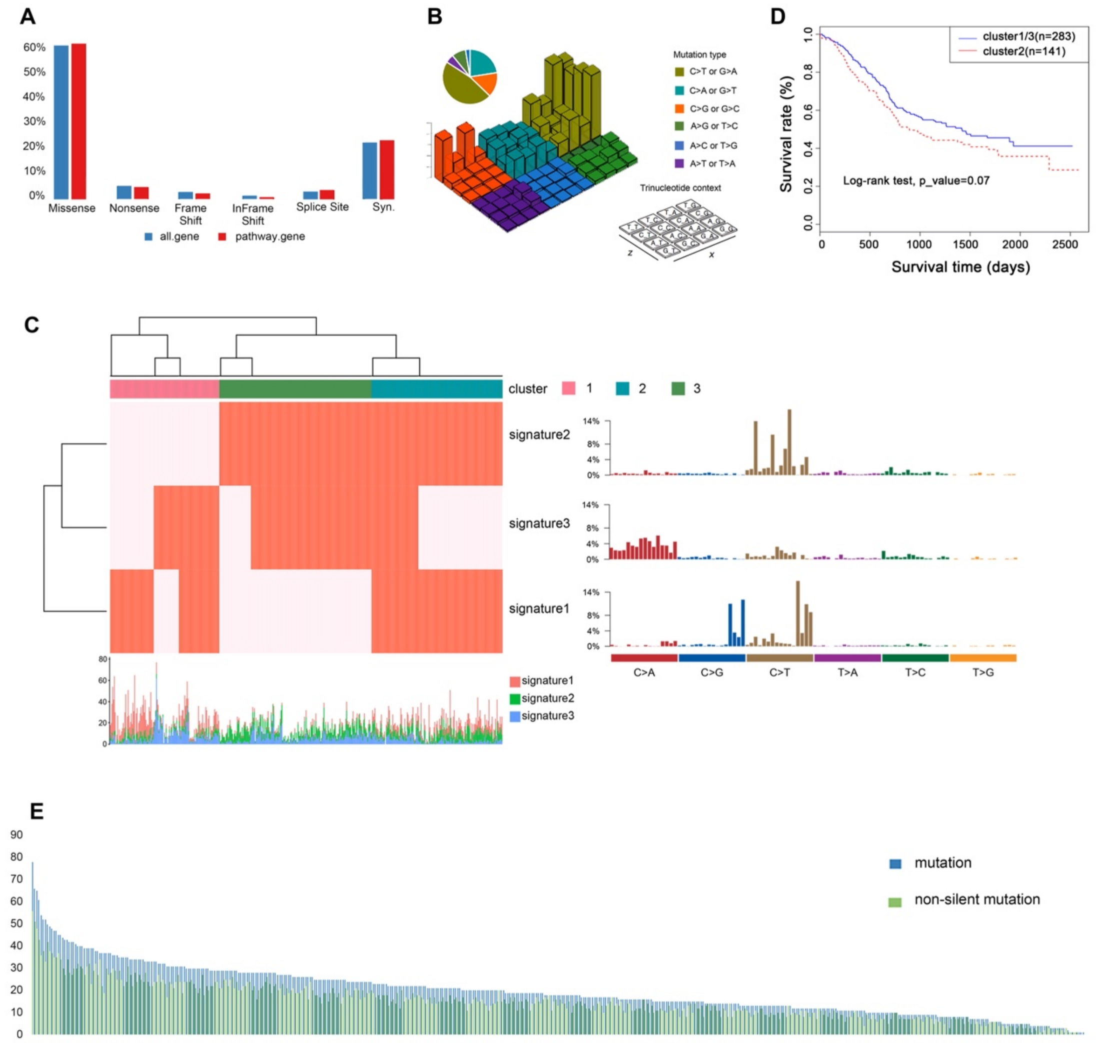

Supplementary figure 1 Mutational signature analysis of ESCC.

(A) Different mutational types of metabolic genes in 490 ESCC patients.

(B) Lego plots of mutational frequencies in the coding regions in ESCC specimens. Base substitutions were classified into six subtypes and each category was represented by different colors. Pie charts represent the distribution of the six subtypes. Base substitutions were further divided into 96 possible mutation types according to the flanking nucleotides surrounding the mutated base.

(C) Heatmap for mutational signatures using sample exposures to one signature identified in ESCC specimens by the NMF method. Each column represents one individual. Each row represents one signature.

(D) Kaplan-Meier survival curves for the three signature-based clusters.

(E) The counts of mutations and non-silent mutations in 490 ESCC patients.

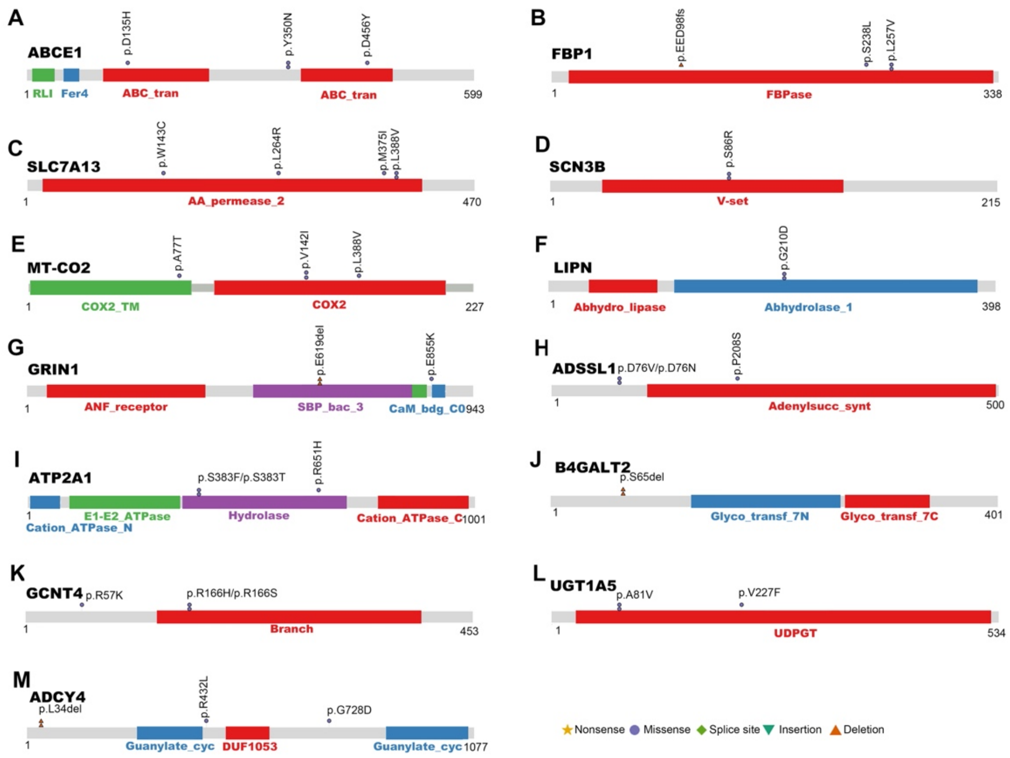

Supplementary figure 2. Recurrent residue enrichment in specific genes.

A schematic representation of the domain structure of proteins (*ABCE1, FBP1, SLC7A13, SCN3B, MT-CO2, LIPN, GRIN1, ADSSL1, ATP2A1, B4GALT2, GCNT4, UGT1A5,* and *ADCY4*) encoded by genes harbored recurrent residues shows the location of somatic variants identified in ESCC tumors.

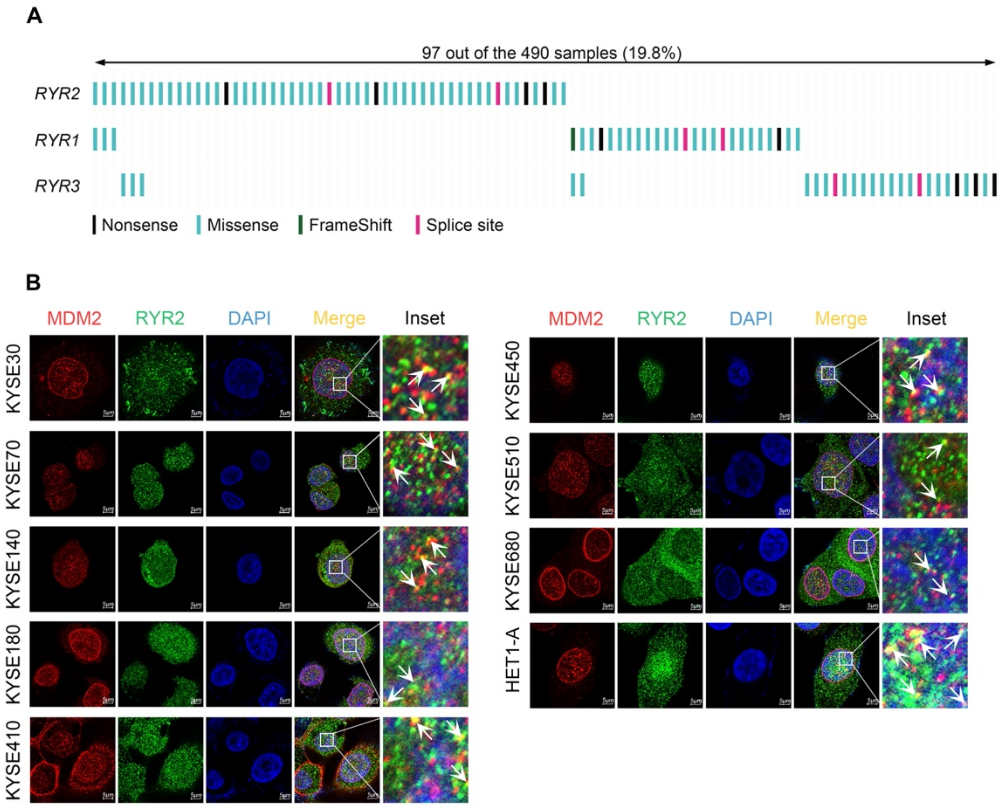

Supplementary figure 3. Mutations of RYR2 and colocalization of RYR2 and MDM2.

(A) Non-silent somatic mutations of *RYR1, RYR2,* and *RYR3.* Narrow bars represent various types of somatic mutations.

(B) Immunofluorescence analysis of RYR2/MDM2 colocalization in KYSE30, KYSE70, KYSE140, KYSE150, KYSE180, KYSE410, KYSE450, KYSE510, COLO680N, and HET-1A cells.

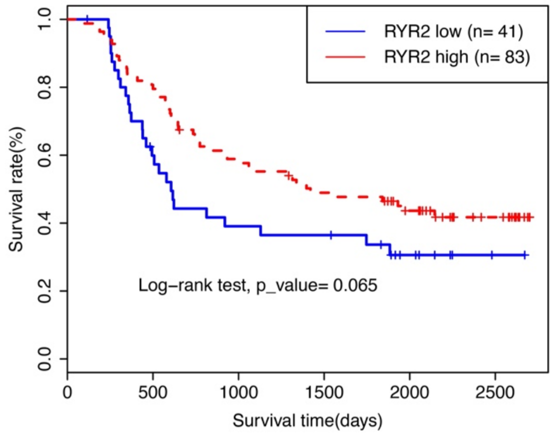

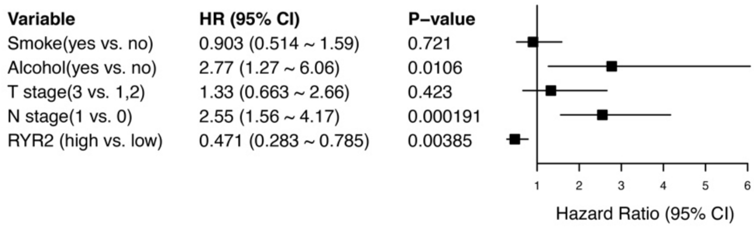

Supplementary figure 4. Association of RYR2 expression levels with patient overall survival (OS) in the Proteomics cohort.

Up: Kaplan-Meier survival analysis of patients with ESCC stratified by RYR2 protein expression levels (n = 124; p = 0.065, log-rank test). Down: Multivariate Cox hazard analysis of RYR2 in an independent cohort.

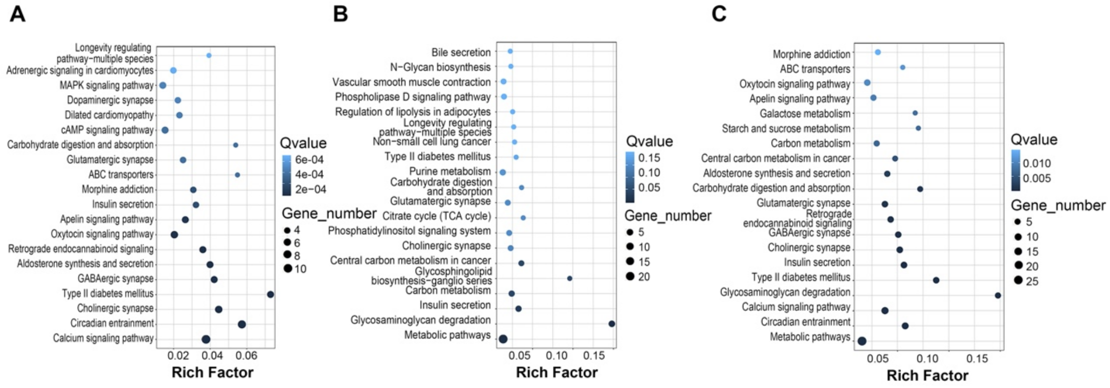

Supplementary figure 5. KEGG pathway enrichment analysis of key genes.

(A) KEGG pathway enrichment analysis of 23 high-frequency mutated genes (non-silent mutation frequency>3%).

(B) KEGG pathway enrichment analysis of genes with CNA over 35%.

(C) KEGG pathway enrichment analysis of key genes mentioned above.

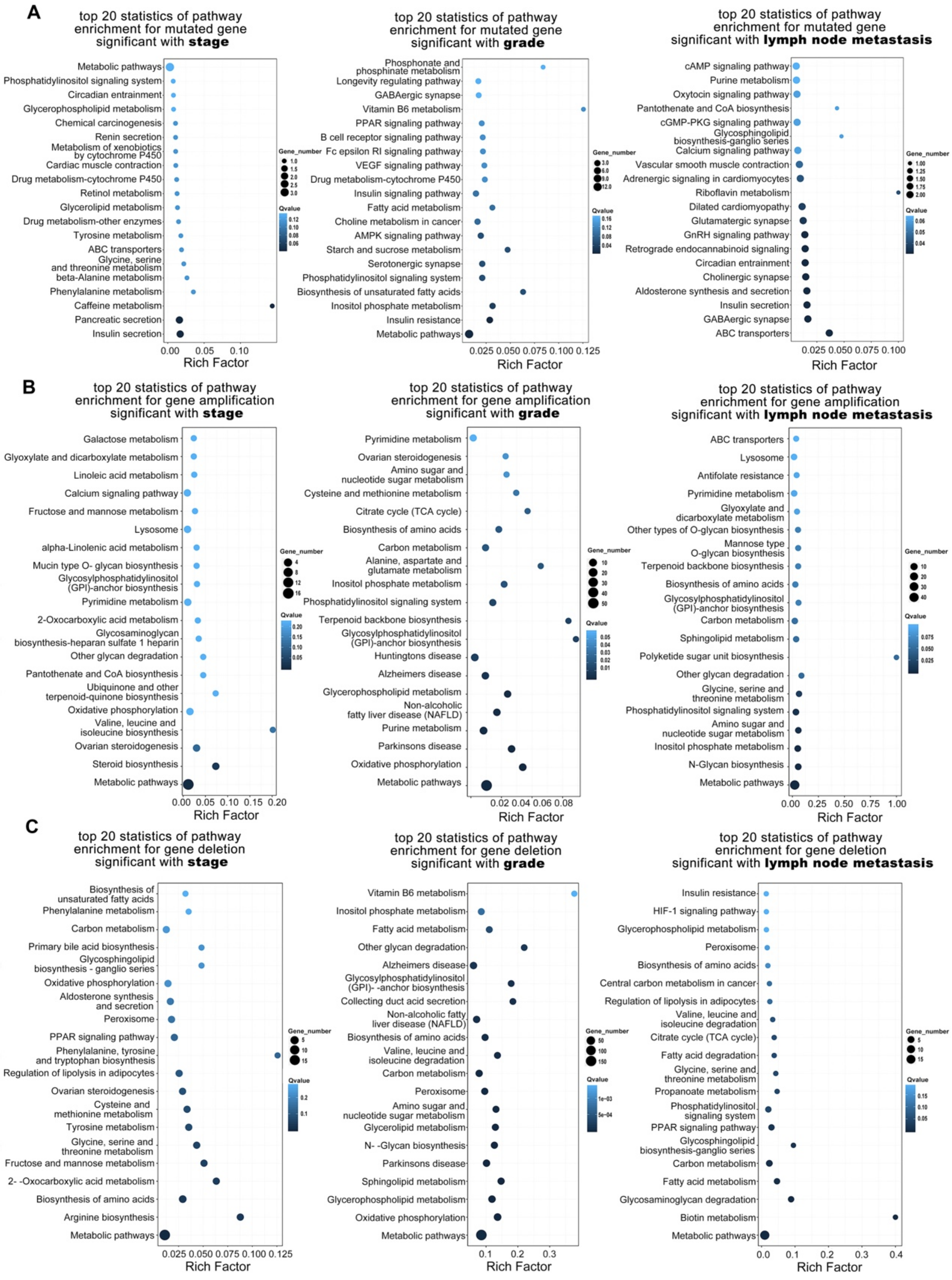

Supplementary figure 6. GO analysis of key genes associated with different pathological factors. Stage (left), grade (middle), and lymph node metastasis (right) significantly associated mutated genes (A), CNA-gain affected genes (B), and CNA-loss affected genes (C) were enriched in different GO categories.

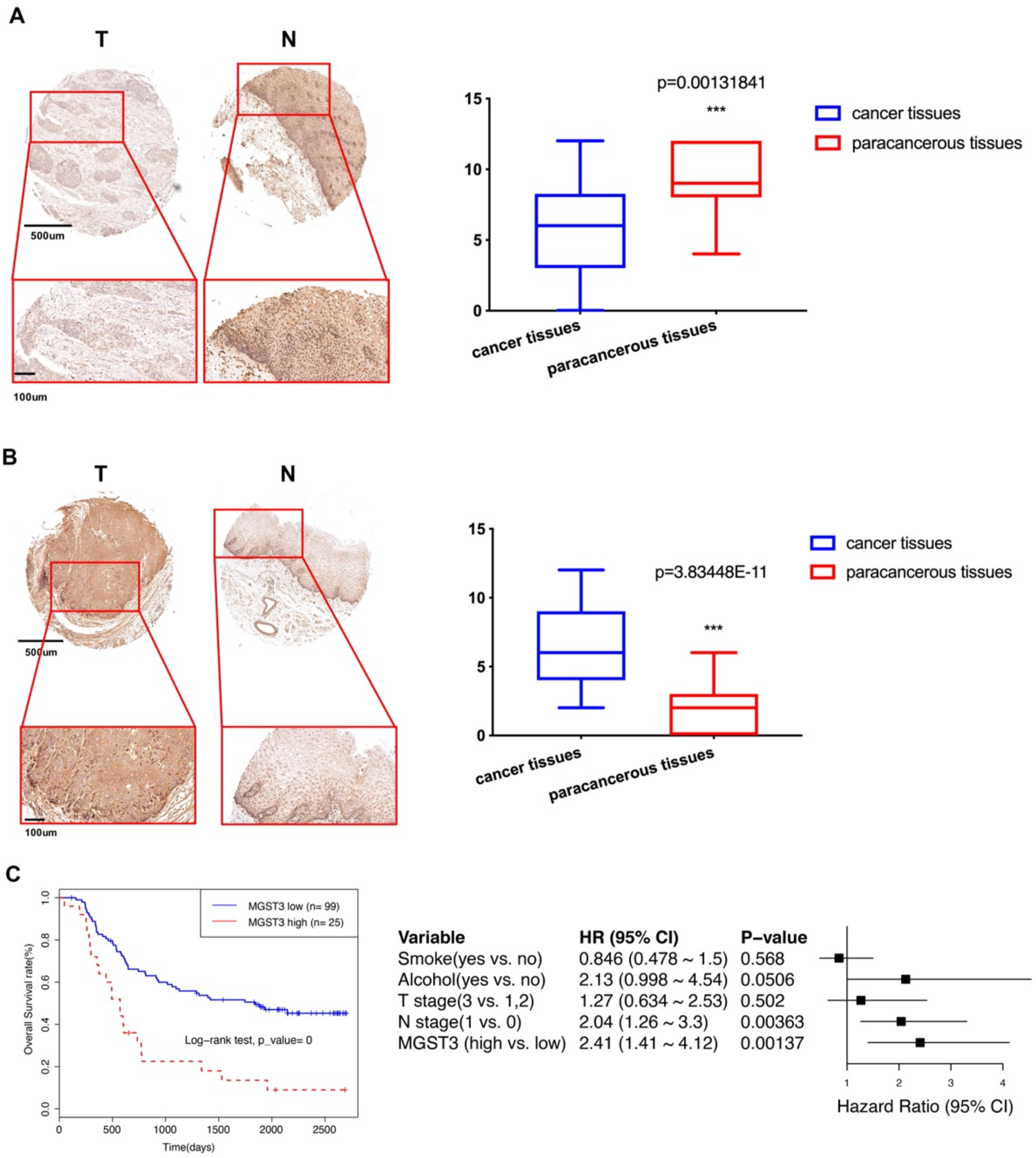

Supplementary figure 7. CYP8B1 and MGST3 serve as potential metabolic driver genes.

(A) Left: Representative images of IHC for CYP8B1 in ESCC tissues (n=46) and paracancerous tissues (n=22). Scale bars, 500 μm. Right: CYP8B1 expression in cancer and paracancerous tissues.

(B) Left: Representative images of IHC for MGST3 in ESCC tissues (n=41) and paracancerous tissues (n=32). Scale bars, 500 μm. Right: MGST3 expression in cancer and paracancerous tissues.

(C) Left: Kaplan-Meier survival analysis of patients with ESCC stratified by MGST3 protein expression levels (n = 124; p = 0, log-rank test). Right: Multivariate analysis of the hazard ratios (HR) showed that the upregulation of MGST3 was a potential metabolic driver gene for the overall survival (by the multivariate Cox proportional hazard regression model). The HR is presented as the means (95% confidence interval, 95% CI).

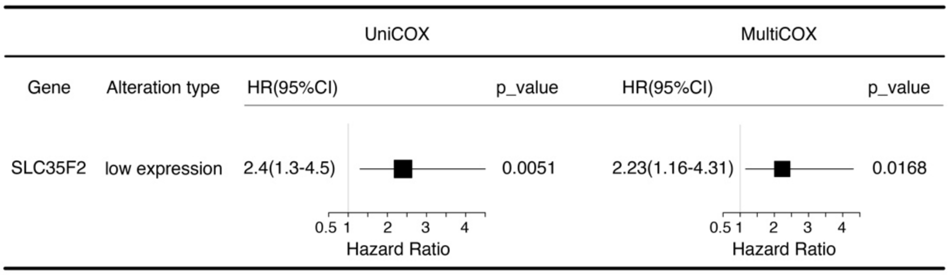

Supplementary figure 8. Prognostic implication of altered metabolic gene *SLC35F2* in the Proteomics Cohort.

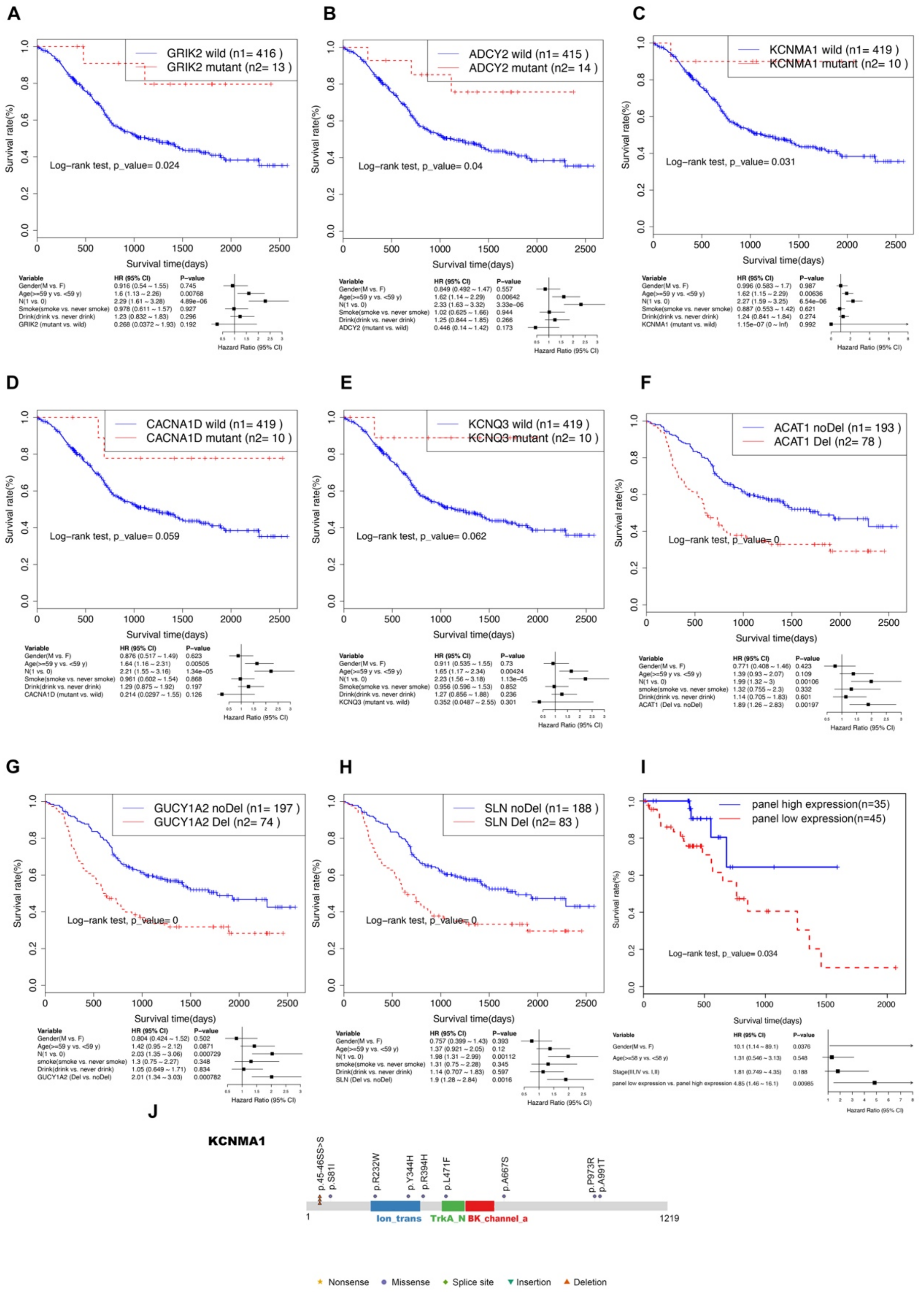

Supplementary figure 9. Clinical significance of altered metabolic genes.

(A) Up: Kaplan–Meier curve of GRIK2-mutated and non-mutated samples. Down: Multivariate Cox regression survival analysis of GRIK2.

(B) Up: Kaplan–Meier curve of ADCY2-mutated and non-mutated samples. Down: Multivariate Cox regression survival analysis of ADCY2.

(C) Up: Kaplan–Meier curve of KCNMA1-mutated and non-mutated samples. Down:

Multivariate Cox regression survival analysis of KCNMA1.

(D) Up: Kaplan–Meier curve of CACNA1D-mutated and non-mutated samples. Down: Multivariate Cox regression survival analysis of CACNA1D.

(E) Up: Kaplan–Meier curve of KCNQ3-mutated and non-mutated samples. Down: Multivariate Cox regression survival analysis of KCNQ3.

(F) Up: Kaplan–Meier curve of ACAT1-deletion and non-deletion samples. Down: Multivariate Cox regression survival analysis of ACAT1.

(G) Up: Kaplan–Meier curve of GUCY1A2-deletion and non-deletion samples. Down: Multivariate Cox regression survival analysis of GUCY1A2.

(H) Up: Kaplan–Meier curve of SLN-deletion and non-deletion samples. Down: Multivariate Cox regression survival analysis of SLN.

(I) Up: Kaplan-Meier survival curve for the CNA-loss affected metabolic gene panel (SLN, ACAT1, and GUCY1A2) in the TCGA Cohort. Down: Multivariate Cox regression survival analysis of CNA-loss affected metabolic gene panel.

(J) A schematic representation of the domain structure of KCNMA1 shows the location of somatic variants identified in ESCC tumors.
